# Single-cell paired-end genome sequencing reveals structural variation per cell cycle

**DOI:** 10.1093/nar/gkt345

**Published:** 2013-04-27

**Authors:** Thierry Voet, Parveen Kumar, Peter Van Loo, Susanna L. Cooke, John Marshall, Meng-Lay Lin, Masoud Zamani Esteki, Niels Van der Aa, Ligia Mateiu, David J. McBride, Graham R. Bignell, Stuart McLaren, Jon Teague, Adam Butler, Keiran Raine, Lucy A. Stebbings, Michael A. Quail, Thomas D’Hooghe, Yves Moreau, P. Andrew Futreal, Michael R. Stratton, Joris R. Vermeesch, Peter J. Campbell

**Affiliations:** ^1^Department of Human Genetics, KU Leuven, Leuven, 3000, Belgium, ^2^Cancer Genome Project, Wellcome Trust Sanger Institute, Hinxton CB10 1SA, UK, ^3^Department of Human Genetics, VIB and KU Leuven, Leuven, 3000, Belgium, ^4^Sequencing R&D, Wellcome Trust Sanger Institute, Hinxton CB10 1SA, UK, ^5^Leuven University Fertility Center, University Hospitals Leuven, Gasthuisberg, Leuven, 3000, Belgium, ^6^Department of Electrical Engineering, KU Leuven, Leuven, 3000, Belgium, ^7^Department of Genomic Medicine, The University of Texas MD Anderson Cancer Center, Houston, Texas, TX 77230-1429, USA, ^8^Department of Haematology, Addenbrooke's Hospital, Cambridge CB2 0QQ, UK and ^9^Department of Haematology, University of Cambridge, Cambridge CB2 2XY, UK

## Abstract

The nature and pace of genome mutation is largely unknown. Because standard methods sequence DNA from populations of cells, the genetic composition of individual cells is lost, *de novo* mutations in cells are concealed within the bulk signal and per cell cycle mutation rates and mechanisms remain elusive. Although single-cell genome analyses could resolve these problems, such analyses are error-prone because of whole-genome amplification (WGA) artefacts and are limited in the types of DNA mutation that can be discerned. We developed methods for paired-end sequence analysis of single-cell WGA products that enable (i) detecting multiple classes of DNA mutation, (ii) distinguishing DNA copy number changes from allelic WGA-amplification artefacts by the discovery of matching aberrantly mapping read pairs among the surfeit of paired-end WGA and mapping artefacts and (iii) delineating the break points and architecture of structural variants. By applying the methods, we capture DNA copy number changes acquired over one cell cycle in breast cancer cells and in blastomeres derived from a human zygote after *in vitro* fertilization. Furthermore, we were able to discover and fine-map a heritable inter-chromosomal rearrangement t(1;16)(p36;p12) by sequencing a single blastomere. The methods will expedite applications in basic genome research and provide a stepping stone to novel approaches for clinical genetic diagnosis.

## INTRODUCTION

Large-scale sequencing of whole-cancer genomes is revealing an unexpectedly diverse array of mutational profiles, hinting at considerable underlying complexity in somatic mutation processes (1–7). However, such studies are necessarily limited by the fact that somatic mutations can only be detected when they have occurred in a lineage of cells that subsequently undergoes significant clonal expansion and is, therefore, already progressing towards malignancy. As a result, questions about the rate of somatic mutation per cell division, the prevalence of mutations in ‘normal’ somatic cells and the influences of carcinogens, ageing or germ line genetic profile on mutation burden cannot be directly answered.

Single-cell genome analysis can bypass these problems (8–17). Recent methods that skim a cell’s genome for DNA copy number alteration yielded new insight in genome mutation during human gametogenesis, embryogenesis and tumorigenesis and in the aetiology of congenital and acquired genetic diseases (9,10,12,13,18). In addition, single-cell genomics is revolutionizing genetic diagnosis of pre-implantation human embryos in the clinic (19–21) and will become increasingly important in cancer diagnosis, prognosis and treatment, allowing analyses of scarce circulating tumour cells (18,22). However, current methods for single-cell analysis have important limitations regarding the accuracy, resolution and the various classes of DNA mutation that can be detected in a cell.

Single-cell whole-genome amplification (WGA) techniques combined with DNA microarray comparative genomic hybridizations or single-nucleotide polymorphism (SNP) array analyses enable the detection of DNA copy number aberrations in a cell’s genome. Unfortunately, even the highest resolution arrays only allow the identification of DNA copy number aberrations that encompass millions of bases in a cell (8–10,18,23–28). The difficulty is to discriminate with confidence DNA copy number aberrations from allelic amplification artefacts induced by the WGA. All WGA methods create random losses or preferential amplifications of alleles that can easily be mistaken for genuine copy number changes by analyses of the signals downstream of WGA. Also DNA structure (29) and nucleotide sequence (13,14,17) artefacts may be introduced but remain largely uncharted for different WGA methods of human cells. Most WGA techniques are underpinned by either an isothermal multiple displacement amplification (MDA) or a polymerase chain reaction (PCR).

Low coverage single-end sequencing of single-nuclei WGA products recently improved the resolution of a cell’s DNA copy number profile by algorithmic focal sequence-read depth analyses (12). However, the authenticity of *de novo* small imbalances detected in a cell remains ambiguous, and inter- or intra-chromosomal structural rearrangements could not be unveiled.

Here, we provide evidence for the detection of three main classes of mutation, including DNA copy number changes, DNA rearrangements and nucleotide zygosity changes, in a single-cell WGA product. Our methods have the potential to discriminate a single-cell copy number variant from an allele drop out or preferential amplification WGA artefact by detecting among the myriad of aberrantly mapping paired-ends induced by the WGA process confirmatory read-pairs across the read-depth anomaly. Application of these methods to cells obtained from an innovative cell culture strategy revealed *de novo* DNA copy number changes acquired within a single cell division. We demonstrate the potential of single-cell paired-end sequencing for detecting structural variants in a cell, including inter-chromosomal rearrangements, which cannot be characterized with existing single-cell methods.

## MATERIALS AND METHODS

### Single-cell isolation

To isolate individual cells related by a cell cycle, one HCC38-cell was plated per 4-cm diameter dish in 3 ml of conditioned medium using RPMI-1640 supplemented with 10% fetal bovine serum (FBS) and 1× PenStrep as a basic medium. Each cell per plate was monitored for attachment and division by light microscopy. On division, trypsin was added to the culture dish to detach both daughter cells. Cells were individually picked using a 0.75-µm Stripper pipette in a volume of 0.5 µl (RPMI-1640; 10% FBS) and placed in a 5-µl droplet of RPMI-1640 supplemented with 10% FBS and 0.0075 mg/ml of bovine serum albumin (BSA) to avoid subsequent adhesion of the cell to the dish. Cells were serially washed in minimum three droplets of 5 µl 1× phosphate-buffered saline and immediately transferred to lysis buffer (see later in the text). All single tumour cells were derived from the HCC38-subclone B8FF4C but were multiple population doublings remote from the original batch of B8FF4C-cells used for DNA isolation to perform standard paired-end sequencing of non-WGA DNA (see later in the text). Single-blastomere cells from *in vitro* fertilized embryos were isolated as described previously (9).

### Subclones

B8F and A6G are two single-cell–derived subclones of the HCC38 breast cancer cell line and were expanded for ∼30 population doublings. Of B8F single-cell–derived subclones, B8FF4C and B8FB3A were obtained. Of A6G single-cell–derived subclones, A6GD7A and A6GE4F were founded. These single-cell–derived subclones were expanded for ∼30 population doublings. Their genomes were characterized by paired-end sequence analysis (Supplementary Table S1) of non-WGA DNA extracted from millions of cells.

### Human blastomeres

The three blastomeres (‘mda-sc1113’, ‘mda-sc1116’ and ‘mda-sc1117’), which were applied for the detection of *de novo* chromosome rearrangement using single-cell sequencing and data analysis, were derived from a 10-cell biopsied human cleavage stage embryo in the evening of Day 3 post-fertilization (9). This *in vitro* fertilized embryo was fresh (not cryopreserved) and classified as good quality (the embryo carried four blastomeres on Day 2 after fertilization, nine blastomeres in the morning of Day 3 after fertilization, <20% fragmentation and equal-sized blastomeres), but it was selected against following pre-implantation genetic diagnosis (PGD, two cells were biopsied for PGD in the morning of Day 3 after fertilization when the embryo consisted of nine cells). The embryo was derived from a couple having a maternal age <35 years, a normal conventional karyotype in both partners, a maternal body mass index within the range of 18–30, initial normal semen parameters according to World Health Organization regulation, no recurrent miscarriages and the couple entered the *in vitro* fertilization and pre-implantation genetic diagnosis (IVF-PGD) programme for a familial microdeletion. The three blastomeres (‘mda-sc1113’, ‘mda-sc1116’ and ‘mda-sc1117’) were whole-genome amplified using MDA (9). The blastomere sequence data, in which the t(1;16)(p36;p12) break point was mapped, were derived from a single cell ‘mda-sc124’ that was biopsied from a human embryo on Day 4 post-fertilization. The embryo was derived from a couple opting for pre-implantation genetic diagnosis because the male partner carried a balanced translocation t(1;16)(p36;p12). In the IVF-PGD cycle, the embryo was not biopsied on Day 3 for fluorescence *in situ* hybridization (FISH)-based pre-implantation genetic diagnosis because of insufficient quality of the embryo (i.e. the embryo consisted of three cells on Day 3 after fertilization and six cells on Day 4 after fertilization; one biopsied cell of this 4-day-old embryo underwent MDA WGA, paired-end sequence and PCR-analysis—see later). The female partner of the couple was aged <35 years, had a normal karyotype and a body mass index within the 18–30 range. The male partner of the couple was carrier of a balanced translocation t(1;16)(p36;p12) of which the break point was unmapped and received a diagnosis of oligoasthenoteratozoospermia (OAT); IVF was performed using ICSI (intracytoplasmic sperm injection).

### Whole-genome amplification

Cells before MDA WGA were isolated in 1.5 µl of lysis buffer (200 mM KOH and 50 mM Dithiothreitol (DTT)). Lysis and MDA were performed as described previously (9,30) using MDA reagents from GE Healthcare. PicoPlex single-cell lysis and amplification were performed according to manufacturer’s instructions with slight modifications (PicoPlex-NGS WGA, Rubicon Genomics, see later in the text).

### Paired-end library preparation

Single-cell MDA products and non-WGA DNA extracted from the HCC38 subclones were sheared using adaptive focused acoustics technology (Covaris Inc.) such that the bulk of the fragments ranged from >200 to ∼600 bp in size. Paired-end sequencing libraries were prepared as described previously (31–33). Libraries were sequenced from both ends for 37 cycles on Illumina GAII or 50 cycles on HiSeq2000 devices (Supplementary Table S1).

Amplification products resulting from the PCR-based PicoPlex WGA method were not sheared or size selected. The libraries were paired-end sequenced on Illumina HiSeq2000 devices for 75 cycles (Supplementary Table S1). The first 12 bases of each read were removed according to manufacturer’s instructions.

Reads were aligned to the reference human genome (GRCh37) using Burrows Wheeler Alignment (BWA) (34). PCR duplicates were removed with Picard (http://picard.sourceforge.net/). Genome coverages were determined by BEDtools (35), and the Genome Analysis Tool kit (GATK—http://www.broadinstitute.org/).

### Single-cell copy number profiling

Focal depths of mapped reads (minimum mapping quality of 30) in a sliding window of 10 kb (making jumps of 5 kb) across the genome were computed for both the single cell and a deep-sequenced non-WGA reference genome using Samtools (36) and CNVseq (37). In those analyses that use windows of 50 kb, a new 50-kb bin was defined every 25 kb. The non-WGA reference genome sequences were derived from germ line DNA extracted from multiple white blood cells (PD4198b, 2 × 108 bp paired-end sequencing, 145 Gb sequenced, 125 Gb mapped, >38× coverage) (6). Bins with count zero in both the reference and the single cell were discarded, as well as bins with a %GC-content of <28%. The GC-content per focal bin was determined using the Genome Analysis Tool kit (GATK—http://www.broadinstitute.org/). Subsequently, the logR ratio of the single-cell focal depth versus the non-WGA PD4198b reference depth signal was computed and normalized for %GC-content using a Loess-fit in R. The logR was further normalized according to the median of the genome-wide logR values. Corrected logR values were segmented using piecewise constant fitting (PCF), which fits a piecewise constant function to the data, controlling the number of change points by a penalty parameter γ (38). In this study, we used a γ-value of 25 unless mentioned otherwise. For the identification of small DNA copy number variants encompassed by a limited number of genomic bins in the multi-cell and single-cell sample γ-values of 5 and 10 were applied. Integer DNA copy number (both before and after segmentation) was estimated as *2^logR^.Ψ*, where the average ploidy Ψ of the cell was estimated based on the logR value of a large reference region with known DNA copy number without large copy number aberrations. The scripts are available on request.

### Rearrangement profiling

Paired-end maps were generated using a new in-house algorithm that will be published separately (J. Marshall *et al.*, manuscript in preparation). Briefly, discordantly mapped read pairs were filtered against BWA read pile-up loci, repeat features and mitochondrial sequences in GRCh37. Additionally alternative mapping locations were evaluated to assess whether both reads could be aligned to an alternative location as a concordant pair. Remaining discordant read pairs were clustered to generate a putative list of rearrangements with respect to the GRCh37 reference genome. Candidate rearrangements found in deep-sequenced normal blood DNA analyses, or previously confirmed by PCR to be germ line in other studies, including the HCC38-matched normal DNA, were removed. These steps produced a paired-end map cured from the majority of the artefacts resulting from BWA-mapping and from putative germ line variants.

For the specificity analyses, these refined paired-end maps were processed further. A reference paired-end map of the non-WGA B8FF4C subclone was computed using R to compare the single-cell paired-end maps with. This reference paired-end map consisted of rearrangements that were spanned by at least two discordantly mapping read pairs in the B8FF4C-refined paired-end map and that encompassed at least 5000 bases (except for inter-chromosomal signatures). Positive predictive values of single-cell paired-end maps were subsequently computed as the amount of true positive single-cell rearrangements (which were defined as discordantly mapping read pair signatures in the single-cell–refined paired-end map that had a match with the reference B8FF4C-refined paired-end map) divided by the total number of rearrangement signatures in the single-cell–refined paired-end map. Per cell, a set of positive predictive values (one for each threshold, see later in the text) was calculated using refined paired-end maps containing only rearrangement signatures supported by a minimum amount of discordantly mapping read pairs, which spanned >5 kb. The thresholds on these minimum amounts were varied from 2 to 20 for each cell. For the sensitivity analyses, putative HCC38 rearrangements from aberrantly mapping read pairs of non-WGA DNA sequences were first confirmed by PCR analysis on non-WGA DNA as described previously (33). Subsequently, the fraction of these PCR-validated rearrangements present in the single-cell–refined paired-end maps was calculated in function of thresholds (2–20) on the minimum amount of aberrantly mapping single-cell read pairs that had to corroborate a rearrangement signature.

Integration of logR or copy number profiles with refined paired-end maps of MDA or PicoPlex single-cell sequences was performed using R-scripting, including the design of the informatics filters that apply a physical window around logR break points as bait to retrieve read pairs from the refined paired-end maps.

All scripts are available on request.

To visualize focal read depth and aberrantly mapping read pair clusters, we applied Circos (39).

### SNP profiling

SNP annotation files were downloaded from http://hgdownload.cse.ucsc.edu/goldenPath/hg19/database/ and were curated using a variety of filters available on request. Using the physical positions of the SNPs, pile-ups of mapped reads spanning the SNP were created using Samtools (36). Digital B-allele frequencies (BAFs) and SNP-calling algorithms were developed using perl and R (scripts available on request). The B-allele fraction of a particular SNP was computed as ‘the number of reads incorporating the SNP B-allele/the number of reads spanning the SNP’. Validated mutations detected by sequencing HCC38 were characterized in the cells using a similar approach. Base mismatches were computed using Genome Analysis Tool kit (GATK—http://www.broadinstitute.org/).

### Cloning of the translocation t(1;16)(p36;p12) break points

The positions of the discordantly mapping read pairs and logR changes in the sequences of cell ‘mda-sc124’ were used to infer the approximate break points on the derivative chromosomes der(16) and der(1) of t(1;16)(p36;p12). Unique primers were designed on the 1p and 16p sequences on each side of the estimated break point for both derivative chromosomes der(16) and der(1) (respectively: forward: 5′-CTTCCTAAATTAGTGTGTGGGTGA-3′ and reverse: 5′-TCCAGTCTTCTCAGGTCACG-3′ and forward: 5′-CCCGAGCTGTCTACTGAAGG-3′ and reverse: 5′-ATTTCGATGTTTTTGTGGTTTTCT-3′) and used to amplify across the break points on der(16) and der(1). A primer set proximal to the break point on der(16) was designed to be used as a control PCR (forward: 5′-CGCATGCCTGACTTACAGAA-3′ and reverse: 5′–GACGGGGCACTATCTCATTT-3′). A PCR reaction mix with a total volume of 25 µl was prepared, containing platinum Taq polymerase (Invitrogen), 1.5 mM MgCl_2_, 200 µM of deoxyribonucleotide triphosphates and 0.25 µM primer. The following PCR programme was used: 94°C for 4 min, 30 cycles of ‘94°C for 30 s, 58°C for 30 s, 72°C for 1 min’ and a final extension of 72°C for 7 min. The PCR products were size separated on a 1% agarose gel and were sequenced on an ABI 3100 automated capillary DNA sequencer using the BigDye Terminator v. 3.1 Cycle Sequencing Kit (Applied Biosystems). SNP typing of the affected sibling carrying the derivative 1 chromosome der(1) was performed using the Illumina CytoSNP12-v2.1 microarray platform (Illumina). The SNP-genotypes, BAFs and the logR values were determined by the Illumina Genomestudio software (Illumina). Subsequently, the logR values were further interpreted by PCF using a γ value of 25.

### Ethical approval

Genetic analyses and sequencing of the human blastomere genomes are approved by the local and federal ethical committees (FCE ADV_040-UZ-KUL and FCE ADV_042-UZ-KUL).

## RESULTS

### Genome coverage of single-cell MDA- and PCR-based WGA sequences

**Figure 1.**
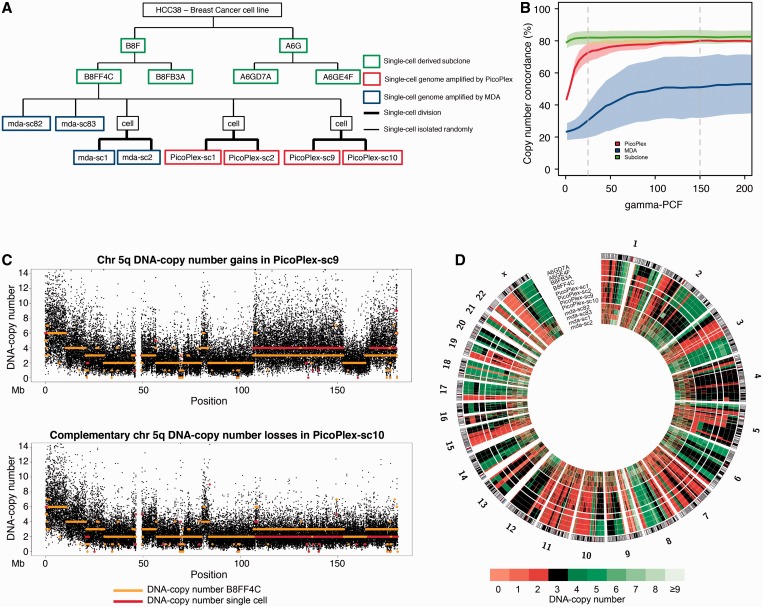
Single-cell DNA copy number profiling by focal read-depth analysis. (**A**) A tree of the single-cell–derived subclones and isolated HCC38-tumour cells. (**B**) Concordances of the DNA copy number profiles of the MDA-WGAed cells (blue), the PicoPlex-WGAed cells (red) and the non-WGAed subclones (green) with the reference B8FF4C copy number profile. The copy number concordance between a sample and B8FF4C was calculated by comparing the copy number states of each 10-kb bin genome wide following focal sequence-depth analyses. The *y*-axis represents the copy number concordance, the *x*-axis the γ penalty parameter of the PCF algorithm used for segmentation (‘Materials and Methods’ section). The mean copy number concordance is depicted as a line, the standard deviation as a shaded region. Two vertical dashed lines indicate the γ values of 25 and 150, respectively. (**C**) Complementary DNA copy number changes on chromosome 5 in two sister cells related by one cell cycle. Orange lines, representing the B8FF4C copy number segments, are overlaid on top of the red lines, which represent the single-cell PicoPlex copy number segments. (Top) Cell ‘PicoPlex-sc9’, (bottom) cell ‘PicoPlex-sc10’. (**D**) Segments of integer DNA copy number states following focal sequence-depth analyses using 10-kb bins and PCF segmentation (γ = 25) across all autosomes and the X chromosome. The integer DNA copy number is depicted as a heat map of which a color legend has been integrated in the figure. The profiles of the non-WGA single-cell–derived subclone samples (A6GD7A, A6GE4F and B8FB3A) and the reference B8FF4C sample are shown, followed by the four PicoPlex-amplified single cells (PicoPlex-sc1, PicoPlex-sc2, PicoPlex-sc9 and PicoPlex-sc10) and the four MDA-amplified single cells (mda-sc82, mda-sc83, mda-sc1 and mda-sc2).

To develop and further study single-cell sequencing methodologies, single cells were isolated from a subclone ‘B8FF4C’, which was characterized by standard paired-end sequence analysis and derived from a cell of the human breast tumour cell line HCC38 (33) (Figure 1A, ‘Materials and Methods’ section). As we envisioned that different WGA approaches may have different, possibly complementary, strengths and weaknesses, we applied both MDA-based and PicoPlex PCR-based WGA technologies to four single-cell genomes (Figure 1A, ‘Materials and Methods’ section). Of each WGA product, a library of paired-end tags was sequenced to maximum 25 Gb (Supplementary Table S1).

Approximately 90% of the sequences resulting from the single-cell MDA products mapped to the reference genome following BWA (34) and covered up to 72% of the human genome dependent on the sequence yield (Supplementary Table S1). Although single-cell PicoPlex WGA products were sequenced deeper (22.8 ± 3.0 versus 13.1 ± 5.4 Gb, Supplementary Table S1), the reads targeted <36% of the human genome and less of the exome in comparison with MDA sequencing (Supplementary Figure S1A–D). In comparison, ∼77% of the genome was covered by ∼12.7 Gb of paired-end sequence of non-WGA DNA of four different HCC38 subclones (Figure 1A, Supplementary Table S1 and Supplementary Figure S1A and B). These data suggest that single-cell WGA results in amplification products that represent only a fraction of a cell’s genome following sequencing, with MDA-based single-cell sequencing attaining a breadth of genomic coverage that is significantly broader than following PicoPlex-based single-cell sequencing (Table 1). Although fewer bases of the genome were covered in single-cell PicoPlex sequences, part of the sequenced loci tended to be covered deeper (Supplementary Figure S1D).

**Table 1.** Performance of single-cell MDA- and PicoPlex-next generation sequencing (NGS)

Performance	Single-cell MDA-NGS			Single-cell PicoPlex-NGS		
Genome coverage	<72%			<36%		
Preferred WGA	+					
DNA copy number by focal read-depth analysis						
Concordance with multi-cell profile	30% (±11%)^a^			72.7% (±6.1%)^a^		
Preferred WGA				+		
DNA structural variation (SV) by paired-end mapping analysis	Signatures with ≥2 supporting read pairs	Signatures with ≥8 supporting read pairs	Signatures with ≥15 supporting read pairs	Signatures with ≥2 supporting read pairs	Signatures with ≥8 supporting read pairs	Signatures with ≥15 supporting read pairs
Sensitivity (max)^b^	60.4% (±13.0%)	27.8% (±12.0%)	11.1% (±7.8%)	39.9% (±2.8%)	15.9% (±0.8%)	9.3% (±1.0%)
Positive predictive values^b^						
Intra-chromosomal SVs						
Deletion	12.4% (±4.8%)	83.3% (±13.6%)	95.0% (±10.0%)	0.2%^b^ (±0.1%)	0.5% (±0.2%)	1.6% (±0.1%)
Tandem duplication	7.7% (±3.4%)	80.3% (±12.4%)	95.0% (±3.6%)	2.2% (±1.1%)	8.5% (±3.3%)	20.5% (±4.9%)
Inversion	0.1% (±0.0%)	2.9% (±1.6%)	8.5% (±3.4%)	0.3% (±0.2%)	0.9% (±0.6%)	1.9% (±1.9%)
Inter-chromosomal SVs:	8.3% (±4.5%)	76.7% (±10.8%)	91.7% (±16.7%)	0.6% (±0.0%)	5.6% (±1.3%)	5.0% (±4.2%)
		
Preferred WGA for integration of the single-cell paired-end map with the copy number profile	+			+		
Preferred WGA for application of filters on the amount of read pairs supporting a putative rearrangement	+			−		
Preferred WGA for rearrangement genotyping	+			±		
Single-nucleotide variation						
Call rate (max)	71.6% (±9.7%)^c^			32.2% (±1.8%)^c^		
Accuracy (max)	87.5% (±4.6%)^d^			84.9% (±2.8%)^d^		
		
Preferred WGA for BAF typing to confirm larger copy number changes	+			+		
Preferred WGA for Mutation genotyping	+			±		

^a^Using PCF penalty γ = 25 (‘Materials and Methods’ section); higher DNA copy number concordances can be achieved by increasing γ (Figure 1B) or genomic bin sizes (Supplementary Figure S4).

^b^Dependent on the aberrantly mapping read pair signature and the number of supporting read pairs (Figure 2).

^c^SNPs covered by two or more reads.

^d^SNPs covered by 20 or more reads.

### Single-cell copy number profiling by analyses of focal sequence depth

We next investigated the performance of both WGA methods for copy number analysis. We derived logR ratios from local sequencing depth using 10-kb windows and normalizing against a deep-sequenced (>30×) non-WGA DNA sample extracted from blood (‘Materials and Methods’ section). Examination of the logR values in the context of %GC-content of the corresponding 10-kb bin revealed a WGA-specific GC bias that was corrected by locally weighted regression (Supplementary Figure S2). Subsequently, logR values were normalized further, segmented by PCF and converted to integer DNA copy number values (‘Materials and Methods’ section).

Concordances of single-cell DNA copy number profiles with the reference B8FF4C landscape were calculated by comparing the copy number states of each 10-kb bin genome wide. Profiles following PicoPlex-WGA single-cell DNA sequencing demonstrated a concordance of 72.7% (±6.1 SD) with the B8FF4C reference (Figure 1B and Table 1). Because in our analyses cells were related by one cell cycle (see later in the text, ‘Materials and Methods’ section), we could unambiguously demonstrate that a considerable proportion of the remaining discordant copy number states was due to the acquisition of novel DNA changes during cell expansion (Figure 1C, see later in the text). As a control, the DNA copy number profiles derived from non-WGA sequences of three single-cell–derived HCC38 subclones (B8FB3A, A6GD7A and A6GE4F) were 82% (±4.4 SD) concordant with the B8FF4C profile corroborating the unstable genetic nature of HCC38 (Supplementary Figure S3 and Figure 1D); hence, the underestimation of the accuracy of the single-cell copy number profiling (Figure 1B).

DNA copy number profiles resulting from single-cell MDA sequences were less accurate and not as reproducible (Figure 1B and D and Table 1). By increasing the stringency of logR segmentation (Figure 1B) or the size of the bins for focal read-depth counting (Supplementary Figure S4A and B), higher genome-wide copy number concordances with B8FF4C could be achieved. However, although PicoPlex-WGA single-cell sequencing considerably outperformed MDA single-cell sequencing for copy number analysis, discriminating bona fide copy number variants from allelic WGA artefacts on the basis of the copy number profile of a single solitary cell remains problematic following both WGA methods.

To investigate the accuracy of the single-cell copy number profiles further, 144 break points of DNA copy number segments ranging from 13 kb to 8.6 Mb detected in B8FF4C by paired-end mapping were scrutinized. In single-cell MDA sequences, 11% (±4.9 SD) of the selected break points demonstrated a copy number match with the segment and the location of the break point within maximum 100-kb distance (Supplementary Figure S5A). The match increased to 21.2% (±2.8 SD) and 75% (±3.9 SD) following single-cell PicoPlex WGA sequencing and non-WGA subclone DNA sequencing, respectively (Supplementary Figure S5A).

Hence, single-cell DNA copy number profiling in isolation is too inaccurate to pinpoint copy number change points, and furthermore, distinguishing true structural DNA imbalances that encompass multiple kilobases or megabases from WGA allele drop out or preferential amplification artefacts on the basis of copy number profiling alone remains problematic regardless of the WGA method used.

### Paired-end mapping increases the accuracy and confidence of single-cell DNA copy number profiling

Read pairs not mapping to the human reference genome as expected point to structural DNA variants. The identification of clusters of such anomalously mapping read pairs in single-cell WGA sequences thus may have the ability to classify sequence read-depth anomalies as allelic WGA-amplification artefacts or bona fide unbalanced DNA rearrangements, and in addition to increase the accuracy of a cell’s copy number landscape.

To test this hypothesis, as well as to identify structural DNA lesions resulting from somatic DNA rearrangement or putative WGA error, the paired-end maps of WGAed cells and non-WGAed subclones were computed and refined by filtering. In this process, raw maps were deprived of false aberrantly mapping read pair signatures resulting from BWA-mapping errors (‘Materials and Methods’ section). These included signatures for putative variants with habitats near repetitive loci, mitochondrial sequences or (other) loci prone to BWA read pile-up, as well as read pairs that may map in a proper pair after adjustment of the mapping conditions. Furthermore, putative germ line structural variants identified across multiple deep-sequenced normal blood samples were discarded as well (‘Materials and Methods’ section). The resulting refined paired-end maps were used in all downstream analyses.

Single-cell paired-end maps were found to be skewed in a manner typical of the WGA method used and revealed at least two orders of magnitude more putative DNA rearrangements when compared with the structure of the reference B8FF4C-tumour genome (Supplementary Figure S6A; on average 133× excess following PicoPlex-WGA, 458× following MDA-WGA). More than 98% of all putative rearrangements spanned by two or more read pairs in the single-cell MDA product resembled genomic inversions and were prominent across all sizes tested (Supplementary Figure S7A and E). In contrast, read pair signatures encompassing putative tandem duplications (54.1%), deletions (6.8%) or inter-chromosomal rearrangements (3.5%) had a higher frequency in the PicoPlex-amplification products when compared with MDA (1.3, 0.3 and 0.1%, respectively), but the vast majority of the tandem duplication artefacts encompassed <5 kb (Supplementary Figure S7B and F). Hence, each WGA method synthesizes many, often specific, chimeric DNA molecules that can be mistaken for genuine structural variants in the cell’s genome following paired-end mapping, making the interpretation of single-cell paired-end maps non-trivial. Using the rearrangement profile of B8FF4C as a reference, we investigated methods for analysing single-cell paired-end sequencing data to distinguish authentic rearrangement signatures from WGA artefacts.

**Figure 2.**
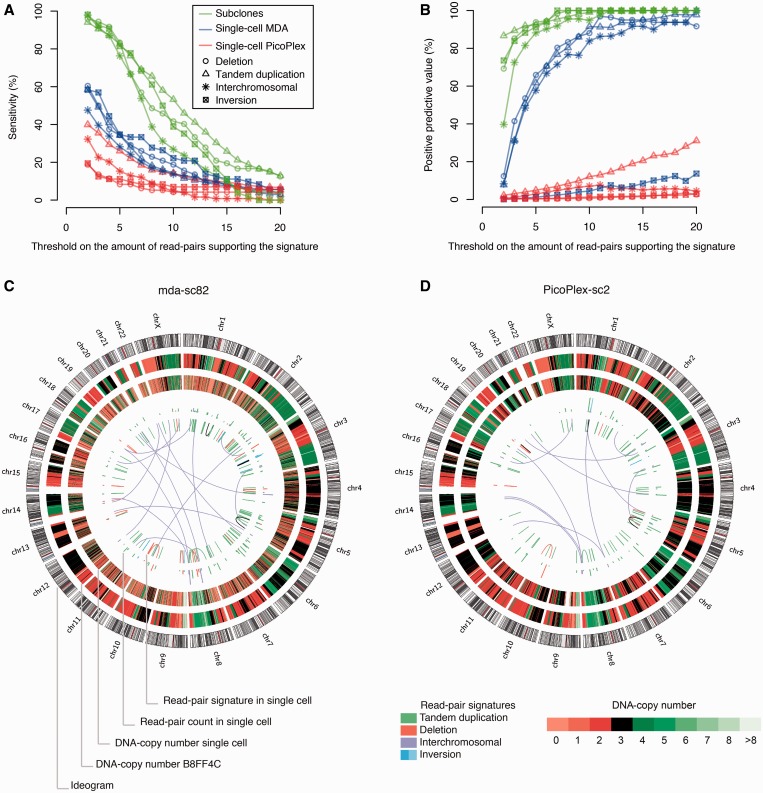
Sensitivity and positive predictive value of single-cell paired-end maps. (**A**) Sensitivity of the single-cell paired-end maps in function of thresholds on the minimum amount of discordant read pairs that had to support a rearrangement signature. A set of 24 deletion-, 124 tandem duplication-, 18 inversion- and 31 inter-chromosomal signatures confirmed by PCR in HCC38 were scored for their presence in the single-cell paired-end maps. The mean sensitivity across the non-WGA subclone, the single-cell MDA and the single-cell PicoPlex paired-end maps are depicted in the *y*-axis. Sensitivities for deletion, tandem duplication, inter-chromosomal rearrangement and inversion signatures are shown separately. For the computations, the refined paired-end maps were used that contained only rearrangement signatures supported by a minimum threshold amount of read pairs (= *x*-axis). (**B**) Positive predictive values of the single-cell paired-end maps for deletion, tandem duplication, inter-chromosomal rearrangement and inversion signatures in function of thresholds on the minimum amount of discordant read pairs that had to support a rearrangement signature. The positive predictive values (= *y*-axis) were computed as the amount of single-cell rearrangements with a matching rearrangement signature in the reference B8FF4C paired-end map, divided by the total number of single-cell rearrangements present in the respective single-cell paired-end map. Refined paired-end maps that contained only rearrangement signatures supported by a minimum threshold-amount of discordant read pairs (= *x*-axis) and which encompassed >5 kb (except for putative inter-chromosomal events) were used for all calculations. The reference B8FF4C paired-end map consisted of signatures that encompassed >5 kb (except for putative inter-chromosomal events) and that were supported by two or more discordantly mapping read pairs. (**C**) A Circos-plot depicting confirmed HCC38 rearrangements identified in single cell ‘mda-sc82’ following paired-end sequencing of the MDA product. From the outside to the inside of the Circos-plot: (i) chromosome ideograms, (ii) the integer DNA copy number heat map (using 10-kb bins and γ = 25) of the non-WGA B8FF4C subclone, (iii) the integer DNA copy number heat map (using 10-kb bins and γ = 25) of the single-cell ‘mda-sc82’ sample, (iv) the amount of read pairs supporting each single-cell rearrangement is depicted by a bar (scale 2–30) at the start of each rearrangement signature and (v) confirmed HCC38 rearrangements identified in single cell ‘mda-sc82’ following paired-end sequencing. Color legends for the rearrangements and the copy number heat map are indicated. (**D**) A Circos-plot depicting confirmed HCC38 rearrangements identified in single cell ‘PicoPlex-sc2’ following paired-end sequencing. From the outside to the inside of the Circos-plot: (i) chromosome ideograms, (ii) the integer DNA copy number heat map (using 10-kb bins and γ = 25) of the non-WGA B8FF4C subclone, (iii) the integer DNA copy number heat map (using 10-kb bins and γ = 25) of the single-cell ‘PicoPlex-sc2’ sample, (iv) the amount of read pairs supporting each single-cell rearrangement is depicted by a bar (scale 2–30) at the start of each rearrangement signature and (v) confirmed HCC38 rearrangements identified in single cell ‘PicoPlex-sc2’ following paired-end sequencing. Color legends for the rearrangements and the copy number heat map are indicated. Circos-plots depicting confirmed HCC38 rearrangements that are identified in all non-WGA subclone and single-cell paired-end maps individually are presented in Supplementary Figure S8.

Analysis of the sensitivity of single-cell paired-end maps indicated that many valid rearrangements are preserved in the single-cell WGA product. Read pair signatures of 24 deletions, 124 tandem duplications, 18 DNA inversions and 31 inter-chromosomal rearrangements, validated by PCR on a non-WGA HCC38 DNA-sample, were scrutinized in the cell’s paired-end maps (Figure 2 and Table 1). On average, up to 60% of the validated structural DNA variants were covered by at least two read pairs following single-cell MDA sequencing (Figure 2A and C and Supplementary Figure S8, depicting all of these rearrangements found in each single-cell paired-end map in Circos-plots). In contrast, PicoPlex WGA sequences on average captured <40% of the validated rearrangements (Figure 2A and D and Supplementary Figure S8).

**Figure 3.**
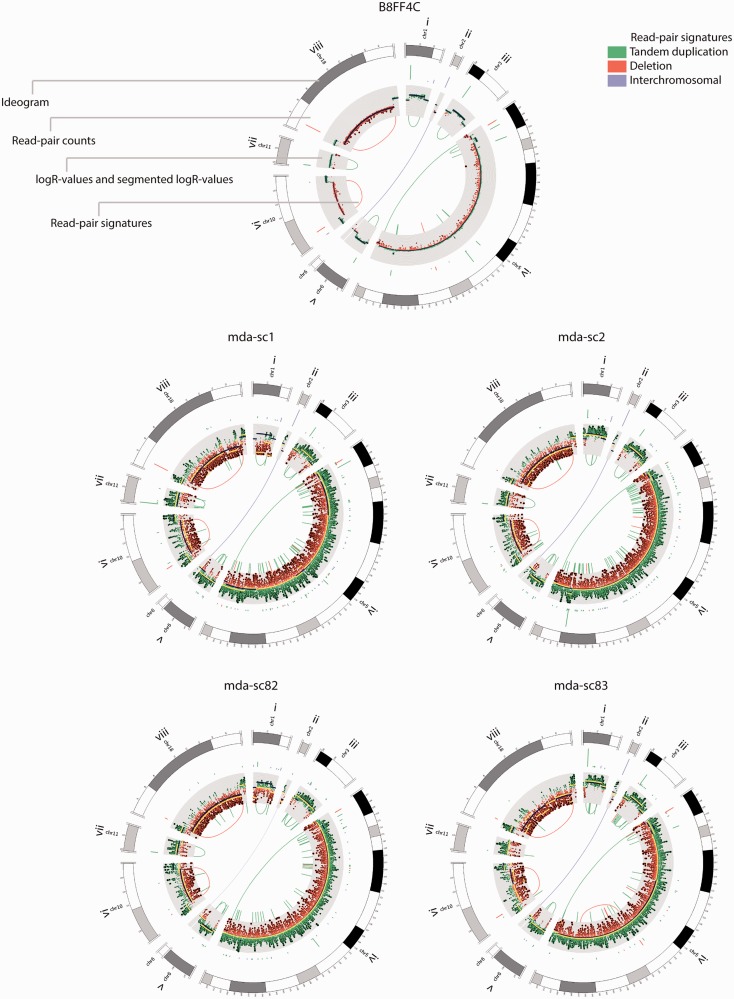
Detection of imbalanced structural variants by paired-end mapping of single-cell MDA-sequences. Integration of focal read-depth anomalies with aberrantly mapping read pairs allows accurate copy number variant detection in single cells and discloses the structure of the DNA imbalances. Read pair signatures typical for tandem duplications, deletions or inter-chromosomal lesions are depicted in the centre of the Circos-plot in green, red and purple, respectively. The amount of read pairs supporting each rearrangement is depicted by a bar (scale 2–30) at the start of each rearrangement signature in the outer circle of the Circos-plot. Subsequently, the logR values are shown on a grid (logR values above zero are depicted in green, below zero in red). Dark blue lines depict the B8FF4C reference logR segments determined from sequences of a non-WGA DNA sample; yellow lines indicate the single-cell MDA logR segments (segmentation penalty γ = 150). The top shows the data of the B8FF4C reference subclone, the bottom four panels depict the single cells ‘mda-sc1’, ‘mda-sc2’, ‘mda-sc82’ and ‘mda-sc83’, respectively. For these samples, the following rearrangements are shown: (i) a 1.7-Mb tandem duplication signature on chromosome 1 (read pair count in the reference = 24, read pair count in the single cells: 3, 14, 8 and 47, respectively). (ii) An inter-chromosomal rearrangement between chromosomes 2 and 6 (a minimum read pair count of nine was applied for putative inter-chromosomal events, if this threshold was not reached a faded purple line represents the rearrangement). (iii) A 1.7-Mb tandem duplication signature on chromosome 3 (read pair count in the reference = 24, read pair count in the single cells: 18, 14, 6 and 21, respectively). (iv) A 46-Mb tandem duplication signature on chromosome 5 (read pair count in the reference = 17, read pair count in the single cells: 9, 6, 4 and 11, respectively). (v) A 1.3-Mb tandem duplication signature on chromosome 6 (read pair count in the reference = 10, read pair count in the single cell: 4, 5, 3 and 4, respectively). (vi) A 4.5-Mb deletion signature on chromosome 10 (read pair count in the reference = 16, read pair count in the single cells: 11, 3, 2 and 12, respectively). (vii) A 1.6-Mb tandem duplication signature on chromosome 11 (read pair count in the reference = 12, read pair count in the single cells: 30, 10, 12 and 0, respectively). (viii) A 8.6-Mb deletion signature on chromosome 18 (read pair count in the reference = 27, read pair count in the single cells: 25, 2, 5 and 5, respectively). Circos-plots for all non-WGA subclone, single-cell MDA and single-cell PicoPlex samples depicting the same loci can be found in Supplementary Figure S9.

This prompted us to explore further the extraction of aberrantly mapping read pairs that support genuine structural variants from the cell’s paired-end map, which is loaded with artefacts because of chimeric DNA molecules of single-cell WGA. Increasing the threshold on the minimum number of aberrantly mapping read pairs that must span a putative rearrangement also increased the positive predictive value of the single-cell MDA paired-end map (Figure 2B and Table 1). On average, >80% of deletion signatures spanned by seven or more read pairs in the single-cell MDA sequences had a matching rearrangement in the B8FF4C reference paired-end map. This approach was effective for deletion, tandem duplication and inter-chromosomal rearrangement signatures, but not for inversions (Figure 2B and Table 1). Furthermore, integrating signatures delineated by multiple anomalously mapping read pairs with single-cell read-depth profiles could pinpoint various structural variants (Figure 3), including deletions and tandem duplications, ranging megabases down to tens of kilobases in size. Genome wide, a set of 72 DNA imbalances in the reference B8FF4C genome, which was corroborated by anomalously mapping read pairs, including deletion and tandem duplication signatures, and which encompassed 13 kb to 8.6 Mb, was selected for further analysis (see Supplementary Figure S5B for a size distribution of these copy number segments). On average, ∼52% of these 72 rearrangements were supported by a cluster of discordantly mapping read pairs in the single-cell MDA paired-end maps. From the latter rearrangements supported by single-cell MDA paired-end mapping, 50% were detected by single-cell relative copy number analysis as well. This is a first methodology that applies anomalously mapping read pair analysis to discriminate DNA copy number variants from likely allele drop out or preferential amplification WGA artefacts in a cell’s MDA product (Figure 3; Supplementary Figures S5B and S9). Furthermore, DNA imbalances resulting from inter-chromosomal rearrangement could be detected in single-cell genomes as well (Figure 2C, Figure 3 and Supplementary Figure S9).

**Figure 4.**
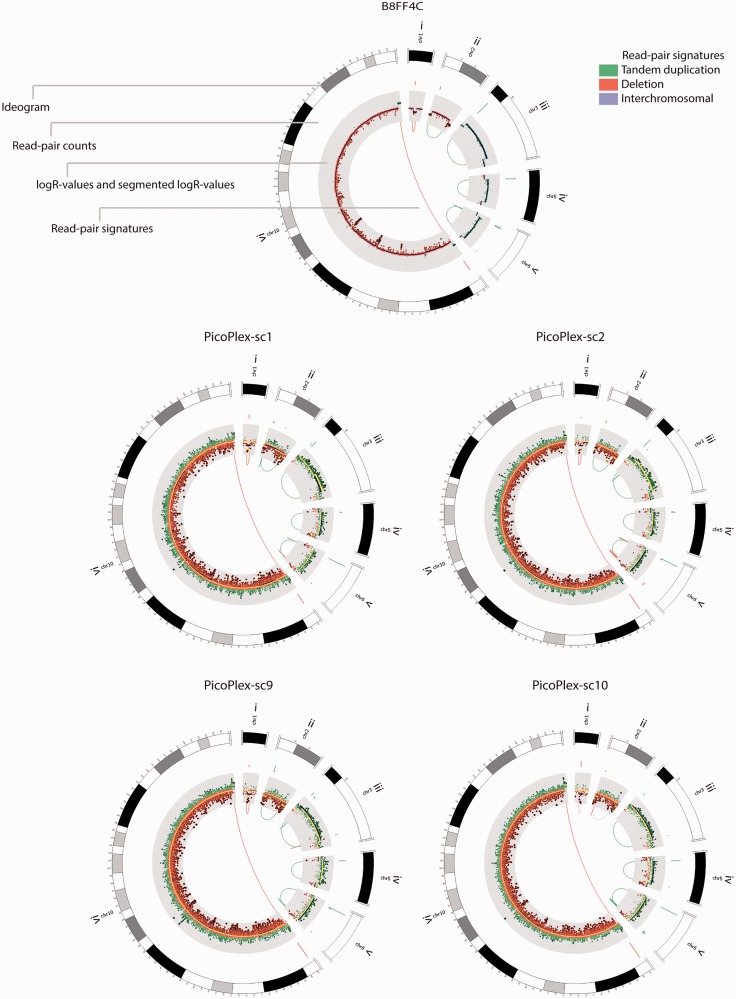
Detection of imbalanced structural variants by paired-end mapping of single-cell PicoPlex-sequences. Aberrantly mapping read pairs typical for tandem-duplication (green), deletion (red) and inter-chromosomal rearrangement (purple) signatures were captured from the refined pool of aberrantly mapping read pairs using a ∼50-kb radius around the single-cell PicoPlex logR break points. For intra-chromosomal rearrangements, only those encompassing >5 kb are depicted in the centre of the Circos-plot. The amount of read pairs supporting each rearrangement is depicted by a bar (scale 2–30) at the start of each rearrangement signature in the outer circle of the Circos-plot. Subsequently, the logR values are shown on a grid (logR values above zero are depicted in green, below zero in red). Dark blue lines depict the B8FF4C reference logR segments determined from sequences of a non-WGA DNA sample, yellow lines the single-cell PicoPlex logR-segments (γ = 150 for the rearrangement on chromosome 10 and γ = 25 for all other rearrangements). The top shows the data of the reference subclone B8FF4C, the bottom four panels depict the single cells ‘PicoPlex-sc1’, ‘PicoPlex-sc2’, ‘PicoPlex-sc9’ and ‘PicoPlex-sc10’, respectively. For these samples, the following rearrangements are shown: (i) a 98-kb deletion signature on chromosome 1 (read pair count in the reference = 6, read pair count in the single cells: 6, 2, 4 and 9, respectively). In PicoPlex-sc10, the discordant read pair signature was present, yet not captured by baiting as the logR segmentation missed the deletion in this cell (shown by a faded red line). (ii) A 2.3-Mb tandem duplication signature on chromosome 2 (read pair count in the reference = 6, read pair count in the single cells: 3, 5, 11 and 1 (shown faded), respectively). (iii) A 1.7-Mb tandem duplication signature on chromosome 3 (read pair count in the reference = 24, read pair count in the single cells: 11, 17, 6 and 4, respectively). (iv) A 1-Mb tandem duplication signature on chromosome 5 (read pair count in the reference = 18, read pair count in the single cell: 5, 7, 10 and 15, respectively). (v) A 1.1-Mb tandem duplication signature on chromosome 6 (read pair count in the reference = 12, read pair count in the single cells: 70, 52, 48 and 83, respectively). (vi) A 62.3-Mb deletion signature on chromosome 10 (read pair count in the reference = 19, read pair count in the single cells: 19, 71, 48 and 59, respectively). The Circos-plots for all non-WGA subclone-, single-cell MDA- and PicoPlex-samples depicting the same loci can be found in Supplementary Figure S10.

Although a read pair count filter was not effective to increase the specificity of single-cell PicoPlex paired-end maps (Figure 2B and Table 1), we investigated whether focal read-depth information could be applied as bait to extract amidst the myriad of WGA paired-end artefacts discordantly mapping read pairs that corroborate the DNA imbalance. As logR segmentation is more sensitive for sequence read-depth anomalies than inferred copy number break points, the physical positions of logR break points plus or minus ∼50 kb were applied as bait. From the same genome-wide set of 72 B8FF4C DNA imbalances as used in the analysis of the single-cell MDA sequences, on average, 30% had a supporting cluster of discordantly mapping read pairs in the single-cell PicoPlex paired-end map, which is in line with the genome coverage breadth and paired-end mapping sensitivity expected following PicoPlex-WGA sequencing (Figure 2A, Supplementary Figure S5B and Supplementary Table S1). Of these discordant read pair clusters, 71% could be efficiently captured by the logR-break point bait, thus discriminating the underlying DNA imbalance from a likely WGA artefact in the cell because the logR anomaly was confirmed by matching aberrantly mapping read pairs (Figure 4 and Supplementary Figure S10). Furthermore, the discordant read pair analyses also captured likely PicoPlex-PCR pile-up WGA artefacts. The latter had the appearance of a real *de novo* DNA gain in a cell’s copy number profile, but the loci were piled with read pair signatures characteristic of tiny flanking tandem duplications (Supplementary Figure S11). At least 4–11 of such false *de novo* DNA gains could be identified per cell (Supplementary Figure S11).

In both single-cell MDA and PicoPlex-amplified genomes, the anomalously mapping read pairs could delineate the break points of the copy number changes with more accuracy than logR segmentation alone (Figures 3 and 4) and allowed the architecture of the copy number change to be identified. The smallest DNA imbalances that were confirmed by both read pair and read-depth analysis and that could be identified in the sequences of MDA- and PicoPlex-amplified cells encompassed ∼14 kb. Furthermore, of a selection of 30 small DNA imbalances confirmed in B8FF4C, including 2 deletion and 28 tandem duplication signatures ranging 13–35 kb, on average 60 and 43% were covered by discordant read pairs following single-cell MDA and PicoPlex sequencing, respectively. Of these rearrangements with discordant read pairs, respectively, 14 and 26% were supported by relative copy number analysis of the single-cell MDA and PicoPlex WGA-products as well.

In conclusion, this methodology, underpinned by single-cell paired-end mapping and novel read pair filters, has the potential to identify a genuine copy number change by the discovery of discordantly mapping read pairs that corroborate the DNA anomaly. Simultaneously, the anomalously mapping read pairs can disclose the architecture of the copy number change and delineate the break points.

### Base pair mutation and SNP genotyping of cells

**Figure 5.**
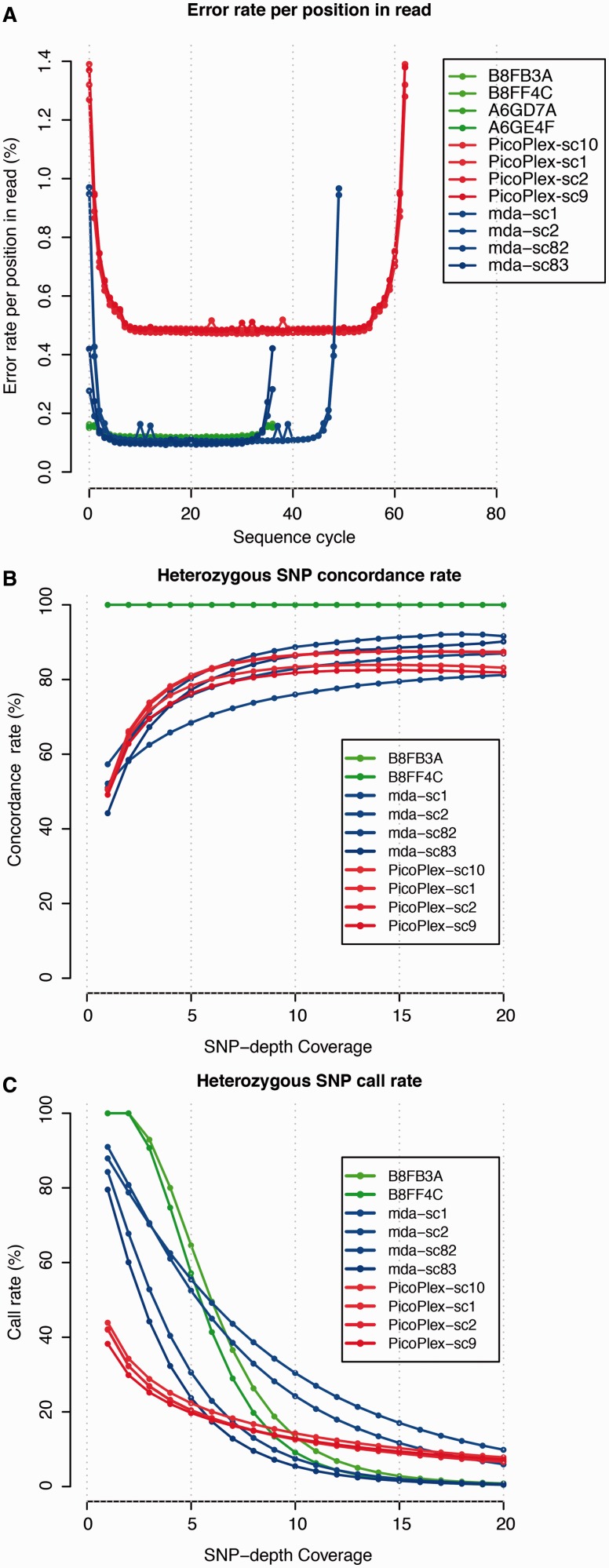
Accuracy of WGA nucleotide copying and genotyping. (**A**) Nucleotide mismatch frequency with the hg19-reference genome at each base of the read. Only bases with a base-call quality of ≥30 in reads having a minimum mapping quality of 30 were considered. It is clear that the PicoPlex WGA method introduces significantly more WGA nucleotide errors than MDA. (**B** and **C**) Approximately 450 000 SNPs, which were heterozygous in the sequences of two HCC38 subclones (B8FF4C and B8FB3A), were genotyped in the single-cell sequences. (B) Single-cell SNP zygosity concordance with the reference genotype (*y*-axis) in function of read depth across the SNPs (*x*-axis). (C) Single-cell SNP call-rate (*y*-axis) in function of read depth across the SNPs (*x*-axis).

Nucleotide substitutions can be identified in single-cell WGA sequences (Table 1). However, as WGA polymerases may not copy every base correctly during the amplification, those errors may be mistaken for genuine nucleotide substitutions in the cell’s genome. To investigate the base fidelity of WGA polymerases, we charted the mismatch frequency of bases (having a base-call quality of ≥30) to the reference genome across the entire length of reads (having a mapping quality of ≥30). This frequency was significantly higher following single-cell PicoPlex-sequencing than following single-cell MDA or non-WGA subclone DNA-sequencing (Figure 5A; two-tailed Kolmogorov–Smirnov test, *P* < 2.2e-16), suggesting that PicoPlex-WGA makes significantly more nucleotide copy errors. To investigate mutation calling in single-cell WGA products further, a set of homozygous (*n* = 6) and heterozygous (*n* = 32) somatic nucleotide substitutions confirmed in HCC38-tumour DNA was genotyped in the single-cell genome sequences. In line with the WGA-specific breadth and depth of sequence coverage, at least quarter of the mutations were covered by multiple reads in the cells and demonstrated zygosity profiles similar to non-WGA DNA sequences (Supplementary Figure S12; on average, 27, 53 and 80% of the heterozygous mutations were covered by at least two reads following single-cell PicoPlex, single-cell MDA and non-WGA subclone sequencing, respectively). To determine the accuracy of heterozygous base-variant typing further, a selection of ∼450 000 SNPs heterozygous in the sequencing data of two HCC38 subclones (B8FF4C and B8FB3A) was evaluated. Increasing sequence depth across the SNP improved the concordance of the single-cell SNP call to that of the reference genotype (Figure 5B and Table 1), but the fraction of SNPs called differed between MDA and PicoPlex single-cell sequences in a manner consistent with the WGA genome coverage (Figure 5C and Table 1).

**Figure 6.**
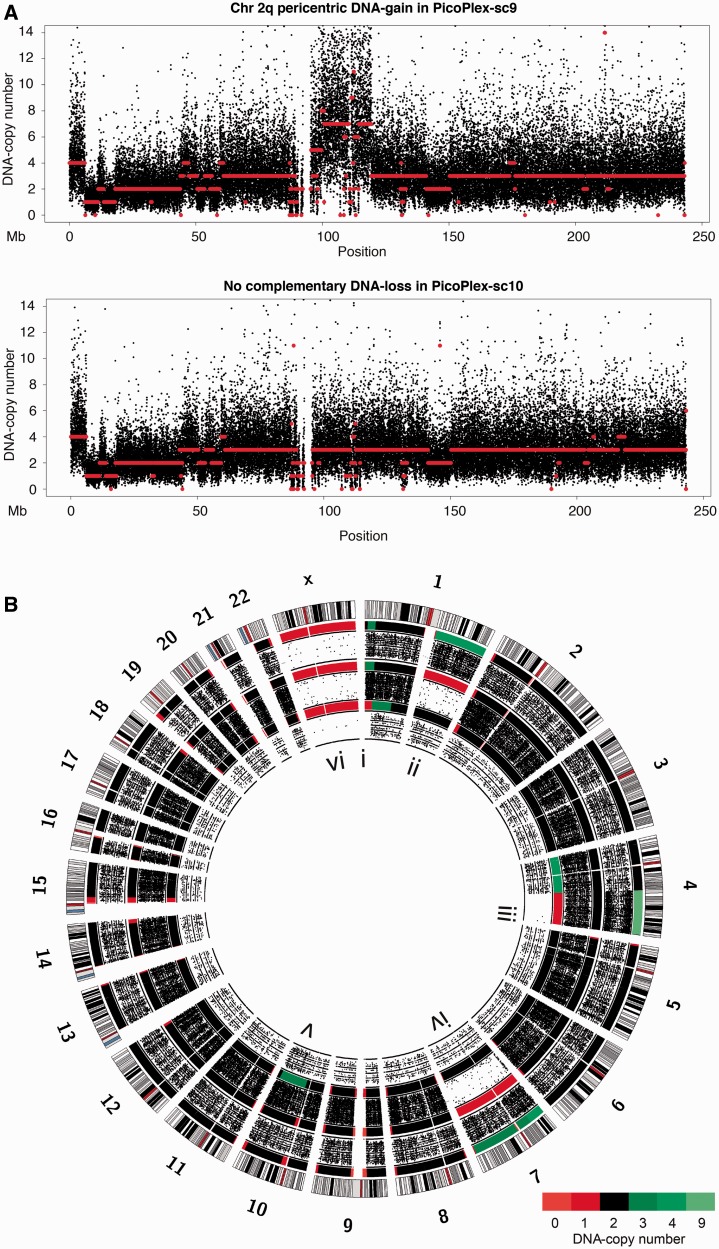
*De novo* structural variants acquired over a single tumour cell cycle and cleavage cell divisions in a human embryo. (**A**) Tumour cells related by one cell cycle. The single-cell genomes were amplified by PicoPlex technology. Chromosome 2 is shown. Single-cell DNA copy number signals are depicted in black and single-cell DNA copy number segments in red. Note that a pericentric DNA gain in cell ‘PicoPlex-sc9’ is not compensated by a deletion in the sister cell ‘PicoPlex-sc10’. (**B**) Genome-wide integer DNA copy number heat maps and BAF of three sister blastomeres of a biopsied human cleavage stage embryo following IVF. The blastomere genomes were amplified by MDA. From the outer to the inner side of the Circos-plot, the DNA copy number heat map and BAF profile of three blastomeres ‘mda-sc1113’, ‘mda-sc1116’ and ‘mda-sc1117’ are shown consecutively. The following *de novo* DNA imbalances were detected across the cell’s genomes (using 50-kb genomic bins for focal read analysis; PCF segmentation penalty γ = 150 for cells ‘mda-sc1113’ and ‘mda-sc1116’; γ = 200 for cell ‘mda-sc1117’, which received lower sequencing coverage; notice that all genuine deletions are corroborated by a loss-of-heterozygosity signature in the BAF): (i) a ∼21-Mb 1pter deletion in blastomere ‘mda-sc1117’ with reciprocal duplications of the same locus in blastomeres ‘mda-sc1113’ and ‘mda-sc1116’. Cell ‘mda-sc1117’ in addition contains a ∼54-Mb duplication flanking the 1pter deletion. (ii) Blastomere ‘mda-sc1116’ carries a 1q-arm deletion with a reciprocal DNA gain in cell ‘mda-sc1113’. (iii) Blastomere ‘mda-sc1117’ has a 4qter deletion with a reciprocal amplification of this locus in cell ‘mda-sc1113’ (notice the clear distortion of the BAF across the nine DNA copies of this locus). The remaining part of chromosome 4 in ‘mda-sc1117’ shows a DNA gain. (iv) Blastomere ‘mda-sc1116’ carries a monosomy 7 with reciprocal trisomy in cell ‘mda-sc1113’. (v) Blastomere ‘mda-sc1117’ carries a 10q-arm duplication. The monosomy X (vi) of this male embryo is detected in all cells. Apparent DNA losses at pericentromeric and telomeric loci, not corroborated by LOH in the BAF (e.g. chromosomes 15 and 19), were interpreted as false positives.

This led us to investigate whether reads encompassing SNPs could be useful for computing digital BAFs across both single-cell MDA and PicoPlex-amplified genomes. Given the breadth and depth of WGA-specific sequence coverage, BAFs across multiple consecutive SNPs over longer distances could be powerful to corroborate bona fide larger structural DNA imbalances in a single-cell sequence. Indeed, in both single-cell MDA and PicoPlex-amplified genomes, BAFs from the low-coverage sequenced cells were able to confirm deletions as well as amplifications in a cell (Figure 6B, see later in the text and Supplementary Figure S13). Deletions resulted in clear patches of loss-of-heterozygosity, whereas copy number amplifications could distort the BAF from the baseline 0.5 ratio (Figure 6B, Supplementary Figure S13).

### Single-cell sequencing enables the detection of copy number changes acquired in a single cell cycle

To investigate the acquisition of *de novo* copy number changes in a single cell cycle, we applied the methods to both G_1_-phase daughter cells (*n* = 6, HCC38) that were derived from a single cell division observed *in vitro*. Remarkably, analysis of the logR of focal depth signals of one cell versus the depth signals of its sister cell revealed clear evidence for genomic alterations acquired in a defined cell cycle. For instance, cell ‘PicoPlex-sc10’ demonstrated putative losses of large genomic loci from one parental copy of the 5q chromosome arm (Figure 1C). The sister cell ‘PicoPlex-sc9’ carried the complementary DNA gains of those loci (Figure 1C and Supplementary Table S2). This unambiguously confirmed that those DNA rearrangements were real. Furthermore, a *de novo* DNA amplification of ∼24 Mb on chromosome 2q flanking the centromere was identified in cell ‘PicoPlex-sc9’ (Figure 6A). Interestingly, this gain was not complemented by a deletion in the sister tumour-cell ‘PicoPlex-sc10’ (Figure 6A). A comprehensive list of the *de novo* DNA imbalances between sister cells is presented in Supplementary Table S2.

To investigate *de novo* rearrangements occurring in a different cell type, we sequenced three sister blastomeres (‘mda-sc1113’, ‘mda-sc1116’ and ‘mda-sc1117’) derived from the same human zygote following *in vitro* fertilization (IVF; ‘Materials and Methods’ section). Numerical and/or structural DNA anomalies are known to occur during cleavage cell cycles of the human zygote following IVF (9,10). Using single-cell MDA sequencing, we detected a terminal DNA amplification of chromosome 4 in blastomere ‘mda-sc1113’, whereas blastomere ‘mda-sc1117’ derived from the same 3-day-old human biopsied embryo (‘Materials and Methods’ section) was found to carry a terminal deletion of the exact same locus and a DNA gain of the remainder of chromosome 4 (Figure 6B). Besides the reciprocity of this rearrangement among both sister blastomeres, also the digital BAFs extracted from the low-coverage sequence confirmed this rearrangement. The genuine 4qter deletion in ‘mda-sc1117’ was supported by loss-of-heterozygosity (LOH) detection in the BAF, whereas the 4qter amplification to a higher DNA copy number count in cell ‘mda-sc1113’ was corroborated by a clear distortion of the BAF across this locus (Figure 6B). In contrast, blastomere ‘mda-sc1116’ was entirely normal for chromosome 4. Additionally, other smaller *de novo* structural rearrangements (e.g. a 1pter deletion and flanking duplication in ‘mda-sc1117’), as well as chromosome-arm imbalances (e.g. a deletion of the 1q-arm in ‘mda-sc1116’) up to whole-chromosome aneuploidies (e.g. a monosomy 7 in ‘mda-sc1116’), could be discovered by single-cell MDA sequencing in this embryo. Each of these DNA anomalies had a reciprocal event in a sister blastomere derived from the same human zygote, confirming these acquired DNA aberrations were real. In addition, BAF analysis further supported the respective genuine DNA deletions clearly. All DNA anomalies are further described in the legend of Figure 6B.

### Single-cell sequencing enables the characterization of an inter-chromosomal rearrangement in a cell of a human cleavage stage embryo

**Figure 7.**
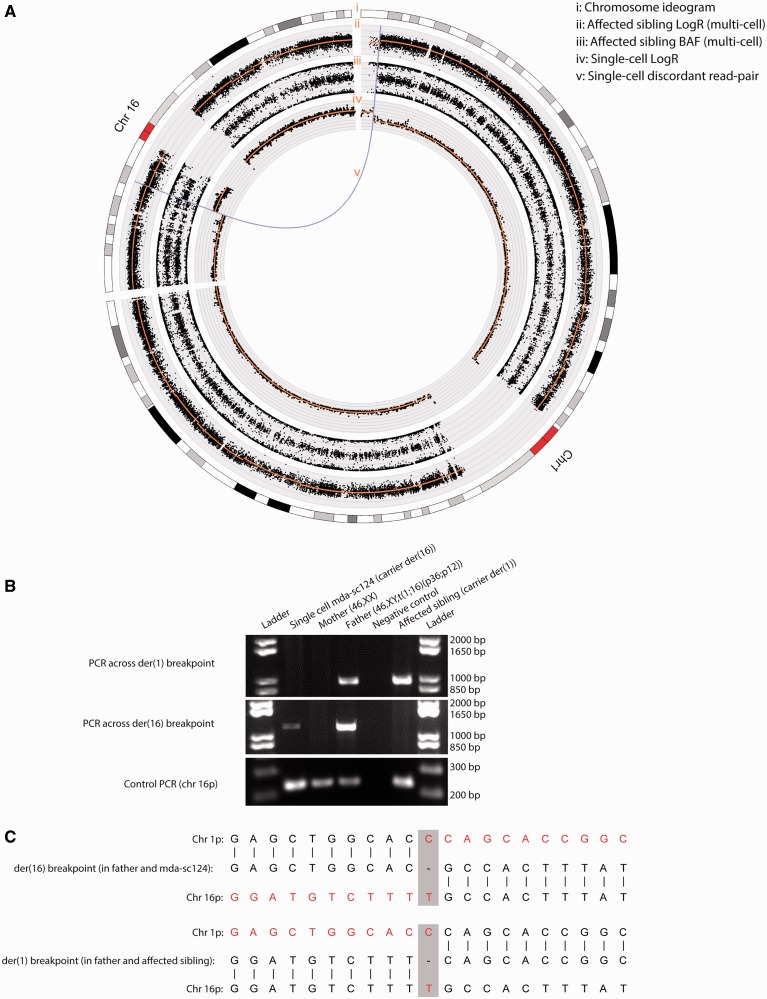
Paired-end sequence analysis of a single cell allows the characterization of an unmapped inter-chromosomal rearrangement to base resolution. By paired-end sequence analysis of a single cell ‘mda-sc124’ biopsied of a human cleavage stage embryo that was derived from a PGD-IVF cycle for a balanced translocation t(1;16)(p36;p12), we were able to pinpoint and characterize the break points on the derivative chromosomes der(1) and der(16) segregating in the family. The male individual of this couple opting for PGD carried the balanced translocation t(1;16)(p36;p12). (**A**) A Circos-plot for the chromosomes 1 and 16 representing (from the outside to the inside): (i) a chromosome ideogram, (ii) the logR values derived from an SNP array analysis performed on the DNA of the affected sibling (γ = 25, orange line), which indicates that the sibling is carrier of the der(1) chromosome, (iii) the BAF derived from the affected sibling’s SNP array analysis supports the DNA imbalances caused by the der(1) in the sibling, (iv) the logR derived from the paired-end sequence data of single cell ‘mda-sc124’ (50-kb bins, PCF segmentation penalty γ = 300, orange line) indicates that cell ‘mda-sc124’ carries the der(16) chromosome and (v) the inter-chromosomal rearrangement read pair signature (purple curve, amount of supporting read pairs = 6) corroborating the der(16) break point in cell ‘mda-sc124’ is shown following single-cell paired-end sequencing and mapping. This single-cell rearrangement is also in line with the der(1) break point in the affected sibling. (**B**) Gel electrophoresis images of the PCR products across the break points. Primers to amplify over the break points were designed based on the paired-end sequence data of the single blastomere ‘mda-sc124’. As expected, the der(16) break point is present in the father carrying the balanced translocation, as well as in cell ‘mda-sc124’, but not in the mother or the affected child. In contrast, the der(1) break point could only be amplified in the father and the affected sibling. A control PCR for a fragment on chromosome 16p confirmed the quality of our DNA samples. (**C**) Capillary sequencing of the PCR products obtained for the single cell ‘mda-sc124’, the father and the affected sibling confirmed the translocation break points to base resolution. At the translocation break point, a single base pair deletion was observed.

To further evaluate the potential of single-cell paired-end mapping, we sequenced a cell biopsied from a human cleavage stage embryo that was derived from a couple opting for PGD because the male partner carried a balanced translocation t(1;16)(p36;p12). The exact break point of the translocation event t(1;16)(p36;p12) was unknown. However, by paired-end sequence analysis of an MDA whole-genome amplified cell (‘mda-sc124’; 3.38 Gb sequenced; 3.19 Gb mapped sequence) biopsied from a human embryo following IVF-PGD (‘Materials and Methods’ section), we were able to characterize the inter-chromosomal rearrangement. In the cell, we detected not only the DNA imbalances resulting from an unbalanced inheritance of the paternal derivative chromosome der(16) but also the matching cluster of discordantly mapping read pairs (*n* = 6) supporting the inter-chromosomal rearrangement t(1;16)(p36;p12) (Figure 7A). Alignment of this single-cell read-depth and read pair data with an SNP array analysis of the DNA of an affected child of the couple subsequently corroborated the correct location of the break points (Figure 7A). Furthermore, by designing a PCR across the break point anticipated from the discordantly mapping read pairs detected in the cell, we could generate the expected amplification products for the derivative chromosome der(16) on the single cell’s WGA-DNA as well as on the father’s DNA, but not using the affected child’s [carrier of the derivative chromosome der(1)] or mother’s DNA as expected (Figure 7B). Similarly, based on the single-cell paired-end map for the der(16) chromosome, we were furthermore able to pinpoint and PCR the reciprocal break point on der(1) carried by the father and the affected child (Figure 7B). Capillary sequencing of the PCR products confirmed the translocation break points to base resolution (Figure 7C). This demonstrates the potential of our single-cell paired-end sequencing approach to characterize structural variants in a solitary cell.

## DISCUSSION

Genome stability remains poorly characterized in both normal and pathological cellular conditions (40,41). To measure DNA mutation rates genome wide over the course of a single defined cell cycle, single-cell genome analysis technologies are required, which are accurate and allow the detection of the full spectrum of genetic variants in a single solitary cell. Unfortunately, such technology does not exist. All methods for single-cell genomics face the difficulty to detect with confidence (*de novo*) DNA copy number and/or single-nucleotide variants in a cell, and thus far, none of the methods has proven the ability to unravel the genomic structure of detected DNA copy number variants (8–10,12,13,16–18,23–28,42–44). Here, we developed methods based on paired-end sequence analysis of single-cell whole-genome amplifications that enabled detecting DNA imbalances of tens of kilobases up to multiple megabases in size, with accurate break point delineation and characterization of the variant’s structural architecture. By sequencing G_1_-phase daughter cells derived from a single cell division, we could demonstrate the acquisition of DNA copy number alterations in one defined cell cycle.

Navin *et al.* (12) demonstrated that low-coverage single-end sequencing of individual nuclei following a PCR-based WGA, and the use of variable genomic bins with a median length of 54 kb for focal read-depth analysis, could increase the resolution of single-cell copy number analyses beyond the level possible with microarray approaches. Although they detected genomic variation between cells, a robust method to distinguish putative WGA artefacts from true copy number variants was lacking. Similarly, separate studies using other single-cell MDA- and/or PCR-based WGA methods applied genomic bin sizes ranging from 500 (13,16) to 200 kb (44) to interpret the single-cell sequences leading to rather low resolution copy number landscapes and in addition did not present independent data sources to confirm observed copy number changes in a cell. We show that single-cell paired-end maps, supplemented with digital allele-frequency profiles, are instructive to discriminate authentic copy number variants (determined using 10-kb genomic bins) from whole-genome amplification artefacts in a single-cell MDA- or PCR-based WGA product. Simultaneously, single-cell paired-end mapping can for the first time reveal the structural architecture of a copy number change in a cell following sequencing of either MDA- or PCR-based single-cell WGA products.

Interpreting single-cell paired-end maps is not straightforward. Both MDA- and PCR-based WGA methods create many chimeric DNA molecules that distort the structure of a cell’s genome in a WGA-specific manner. At least 100 times more putative DNA rearrangements were detected in the cells following our analysis when compared with the structure of the reference B8FF4C-tumour genome. Hence, novel informatics filters were required that sift through the myriad of paired-end artefacts present in the single-cell maps to find valid rearrangements, even if those maps were first filtered for recurrent algorithmic BWA mapping artefacts. The vast majority of aberrantly mapping read pairs (>98%) following MDA single-cell sequencing were characteristic of DNA inversion events, followed by tandem duplication, deletion and inter-chromosomal read pair artefacts. In a mechanistic model, these artefacts were caused by liberating 3′-DNA extending ends from their template, allowing them to anneal to ectopic loci, which is corroborated by sequencing data of single-bacterium MDA-amplified cells (29,45). Interestingly, increasing the threshold for the minimum amount of read pairs that must span a putative deletion, tandem duplication or inter-chromosomal rearrangement in the cell increased the concordance with the reference paired-end map derived from non-WGA DNA, allowing the identification of valid structural variants. This suggests that such artefacts were preferentially instigated late in the MDA reaction with mounting DNA concentration, whereas the kinetics for DNA inversion artefacts were significantly more proficient from the beginning of the MDA reaction. In contrast, in the sequences of the single-cell PCR-based PicoPlex WGA products, valid rearrangements could not be filtered out by a minimum amount of read pairs having to span a putative rearrangement, indicating that most paired-end artefacts were instigated in the first rounds of genome amplification. However, informatics filters using a physical window around logR break points as bait could retrieve read pairs from the paired-end map that suggested focal read-depth anomalies to be bona fide structural DNA imbalances rather than allele drop out or preferential amplification WGA artefacts. Hence, the aberrantly mapping read pairs following both MDA- and PicoPlex-based single-cell paired-end sequencing could corroborate single-cell DNA imbalances, increase the accuracy of the copy number break points estimated in a cell and reveal the architecture of the DNA imbalance. The refined paired-end maps, which are used for integration with DNA copy number profiles, are more sensitive following single-cell MDA sequencing (max ∼60%) than following single-cell PicoPlex sequencing (max ∼40%), which is likely because of the lower representation of the genome after single-cell PicoPlex sequencing. In line with reports that use DNA microarray or SNP array analyses of single-cell MDA- or PCR-based WGA products for copy number profiling of individual cells in research or clinical practice (10,26,46,47), we found that the primary DNA copy number profiles resulting from high-resolution focal sequence read-depth analyses are more accurate following single-cell PicoPlex sequencing than following single-cell MDA sequencing. The natures of the predominant MDA inversion artefacts may be one of the putative causes that distort the copy number profile of a cell’s MDA product.

Xu *et al.* (15) and Hou *et al.* (14) recently investigated subclonal single-nucleotide mutations in cancers by exome sequencing of single-cell MDA products. Also, Zong *et al.* (44) searched for nucleotide changes acquired in cancer cells using full-genome sequencing of single-cell WGA products. However, in all studies, despite using DNA polymerases with proofreading capacity in the WGA reaction, data of at least three cells were required to deliver reliable nucleotide variant calls because of WGA and sequencing errors, thus precluding base mutation calling in a WGA product of a single-multiploid cell. In contrast, MDA products of single haploid cells may be used for *de novo* mutation detection (13). In line with these findings, we show that single-cell MDA and PicoPlex sequences have different nucleotide copy imperfections, but allow genotyping SNPs and point mutations and are powerful for the computation of digital SNP B-allele fractions. Although the single-cell PicoPlex sequences attained a lower genome coverage and SNP-call rate than the single-cell MDA sequences, the SNP B-allele fractions following both WGAs were able to corroborate large structural DNA rearrangements and reveal loci with loss-of-heterozygosity in a single-multiploid cell.

Using our methods, we showed that in a single cell cycle, the reshuffling of pieces of DNA could be observed, allowing us to gain further insight in ongoing chromosome instability in a human cleavage stage embryo and a human breast cancer cell line. By sequencing of individual HCC38 breast cancer cells, we demonstrated that novel DNA gains were accumulated during a cell cycle and showed that an acquired amplification was not necessarily compensated for by a loss in the sister cell. This may suggest that the extra DNA resulted from additional round(s) of DNA replication of that locus. Interestingly, the HCC38 cell line, as well as some other breast tumour cell lines and primary breast tumour cells, contain an unusual high number of tandem duplications (33). In a hypothetical model, the mutator phenotype may be underpinned by a DNA replication error and repair mechanism. In this model, an origin of replication fires more than once on which the extra strand of DNA in the replication fork is resolved as a tandem duplication (48).

In addition, we demonstrate for the first time the ability to pinpoint and characterize the structural architecture of an unmapped inter-chromosomal rearrangement t(1;16)(p36;p12) segregating in a family to base resolution based on paired-end sequence analysis of a single cell of a human embryo. This not only illustrates the strength of our method, but also hints at the putative applicative value of new single-cell paired-end sequencing methods in the clinic in the future. Various research groups have begun to explore the applicative value of next-generation sequencing of pools of cells for clinical genetic testing when only a small amount of cells is available (49,50). For instance, Yin *et al.* (50) sequenced at low-coverage PCR-based WGA products of pools of three to eight trophectoderm cells biopsied from human blastocysts following IVF to generate low-resolution copy number profiles, which enabled them to successfully detect inherited and acquired DNA imbalances encompassing multiple megabases. We hypothesize that application of part of the principles developed in our study, including integrating copy number data with read pair and SNP B-allele fraction analyses, may further improve the resolution, accuracy and reliability of copy number profiles computed from sequences of WGA products of pools of cells as well. Besides applications for pre-implantation genetic diagnosis of human embryos following *in vitro* fertilization, also monitoring and diagnosing cancer in patients by analysing scarce tumour cells circulating in a patient’s blood stream may become feasible. The genetic characterization of such liquid tumour biopsies could in addition provide valuable information to direct therapy over the course of treatment of a patient (22).

In conclusion, we have further shown that single-cell sequencing is a powerful method to study genome mutation in somatic cells. The presented methodology can produce novel understanding of genomic (in)stability to the per cell cycle level in various cell types and processes. These include understanding of the acquisition of genetic changes during induced pluripotent stem cell derivation, the effects of mutagens on a cell cycle and the influences of carcinogens, ageing or germ line genetic profile on general mutation burden. Furthermore, the genetic dissection of normal organs, pre-malignant tissues and established tumours to the single-cell level will provide insights into the operation of fundamental processes of genome maintenance in health and their disruption in cancer. Finally, we anticipate that principles of single-cell paired-end sequencing may eventually contribute to novel clinical applications in molecular diagnosis, such as the analysis of human blastomeres and circulating tumour cells that are often burdened with structural aberrations that cannot be profiled with existing single-cell methods.

## ACCESSION NUMBERS

The data have been submitted to the European Genome-phenome Archive (EGA; https://www.ebi.ac.uk/ega/) and are available through accession number EGAD00001000154.

## SUPPLEMENTARY DATA

Supplementary Data are available at NAR Online: Supplementary Tables 1 and 2 and Supplementary Figures 1–13.

## FUNDING

Agency for Innovation by Science and Technology (IWT) [SBO-60848 to J.R.V.]; Research Foundation Flanders (FWO) [FWO-G.A093.11 to T.V.]; University of Leuven (KU Leuven) SymBioSys [PFV/10/016 to Y.M., J.R.V. and T.V.]. FWO [V.4.140.10.N.00 to T.V.]; P.V.L. is a postdoctoral researcher of the FWO and is supported by a travel grant from the FWO. N.V.D.A. is a PhD student supported by the FWO [1.1.H.28.12]. P.K. is a PhD student supported by KU Leuven SymBioSys [PFV/10/016]. M.Z.E. is a PhD student supported by IWT [TBM-090878 to J.R.V., T.V., Y.M. and T.D.]. L.M. is supported by KU Leuven [CREA/11/023]. Funding for open access charge: Research Foundation Flanders FWO (Belgium) [FWO G.A093.11 to T.V.].

*Conflict of interest statement*. None declared.

## Supplementary Material

Supplementary Data
