# Supplementary material for: Single-cell paired-end genome sequencing reveals structural variation per cell cycle
Source: Nucleic Acids Res. 2013 Apr 27;41(12):6119–38. doi: 10.1093/nar/gkt345 (PMC3695511; doi:10.1093/nar/gkt345)
Supplement: Supplementary Data [file supp_41_12_6119__index.html]

Single-cell paired-end genome sequencing reveals structural variation per cell cycle — Supplementary Data 

# Single-cell paired-end genome sequencing reveals structural variation per cell cycle

## Supplementary Data

files

**Files in this Data Supplement:**

- Supplementary Data - pdf file
