# Supplementary material for: Single-cell paired-end genome sequencing reveals structural variation per cell cycle
Source: Nucleic Acids Res. 2013 Apr 27;41(12):6119–38. doi: 10.1093/nar/gkt345 (PMC3695511; doi:10.1093/nar/gkt345)
Supplement: Supplementary Data [file supp_gkt345_nar-00534-h-2013-File009.pdf]

Supplementary Table 1

Supplementary Table 1: General sequence and genome coverage information

Tissue	WGA	Sample	Read length (bp)	Bases sequenced (Gb)	Bases mapped (Gb)	Average Insert size (bp)	Genome Coverage (%) **
HCC38-tumor cell	MDA	mda-sc82	37 + 50	8.69	7.98	495	63.0
HCC38-tumor cell	MDA	mda-sc83	37 + 50	8.19	7.49	550	60.2
HCC38-tumor cell	MDA	mda-sc1	50	18.81	17.11	285	67.3
HCC38-tumor cell	MDA	mda-sc2	50	16.63	15.22	285	72.2
HCC38-tumor cell	PicoPlex	PicoPlex-sc9	75	25.22	20.29	225	31.1
HCC38-tumor cell	PicoPlex	PicoPlex-sc10	75	25.57	20.35	225	35.8
HCC38-tumor cell	PicoPlex	PicoPlex-sc1	75	20.12	17.14	225	35.3
HCC38-tumor cell	PicoPlex	PicoPlex-sc2	75	20.46	17.41	225	35.0
Blastomere	MDA	mda-sc1113	50	9.35	8.57	375	65.3
Blastomere	MDA	mda-sc1116	50	17.37	15.78	360 and 165 *	74.1
Blastomere	MDA	mda-sc1117	50	3.02	2.68	390	40.5
HCC38-subclone	-	B8FF4C	37	11.43	10.26	400	76.6
HCC38-subclone	-	A6GD7A	37	13.12	11.75	360	78.7
HCC38-subclone	-	A6GE4F	37	13.07	11.55	430	76.6
HCC38-subclone	-	B8FB3A	37	13.33	11.84	380	77.9

* two libraries were sequenced

** genome coverages were calculated with reads having a minimum mapping quality of 30

Supplementary Table 2

Supplementary Table 2: Identified de novo genetic aberrations

Cells:	PicoPlex-sc9	PicoPlex-sc10
De novo aberrations in cell cycle	Chr2q DNA-gain [95.52 Mb – 119.25 Mb]<br>Chr5q DNA-gain [106.79 Mb – 153.13 Mb]<br>Chr5q DNA-gain [164.47 Mb – 180.71 Mb]<br>Chr8q DNA-gain [125.38 Mb – 131.96 Mb]<br>Chr18q DNA-gain [20.23 Mb – 46.66 Mb]<br>Chr18q DNA-gain [55.30 Mb – 78.01 Mb]	Chr5q DNA-loss [106.79 Mb – 153.13 Mb]<br>Chr5q DNA-loss [164.47 Mb – 180.71 Mb]<br>Chr8q DNA-loss [125.38 Mb – 131.96 Mb]<br>Chr18q DNA-loss [20. 23 Mb – 46.66 Mb]<br>Chr18q DNA-loss [55.30 Mb – 78.01 Mb]
Cells:	PicoPlex-sc1	PicoPlex-sc2
De novo aberrations in cell cycle	/	/
Cells:	mda-sc1	mda-sc2
De novo aberrations in cell cycle	Chr1p DNA-loss [66.68 Mb – 114.15 Mb]	Chr4q DNA-gain [118.95 Mb – 191.03 Mb]<br>Chr12q DNA-gain [45.61 Mb – 133.83 Mb]
Cells:	mda-sc82	mda-sc83
De novo aberration in comparison with B8FF4C	Chr1p DNA-loss [87.32 Mb – 121.45 Mb]	/

Supplementary Figure 1

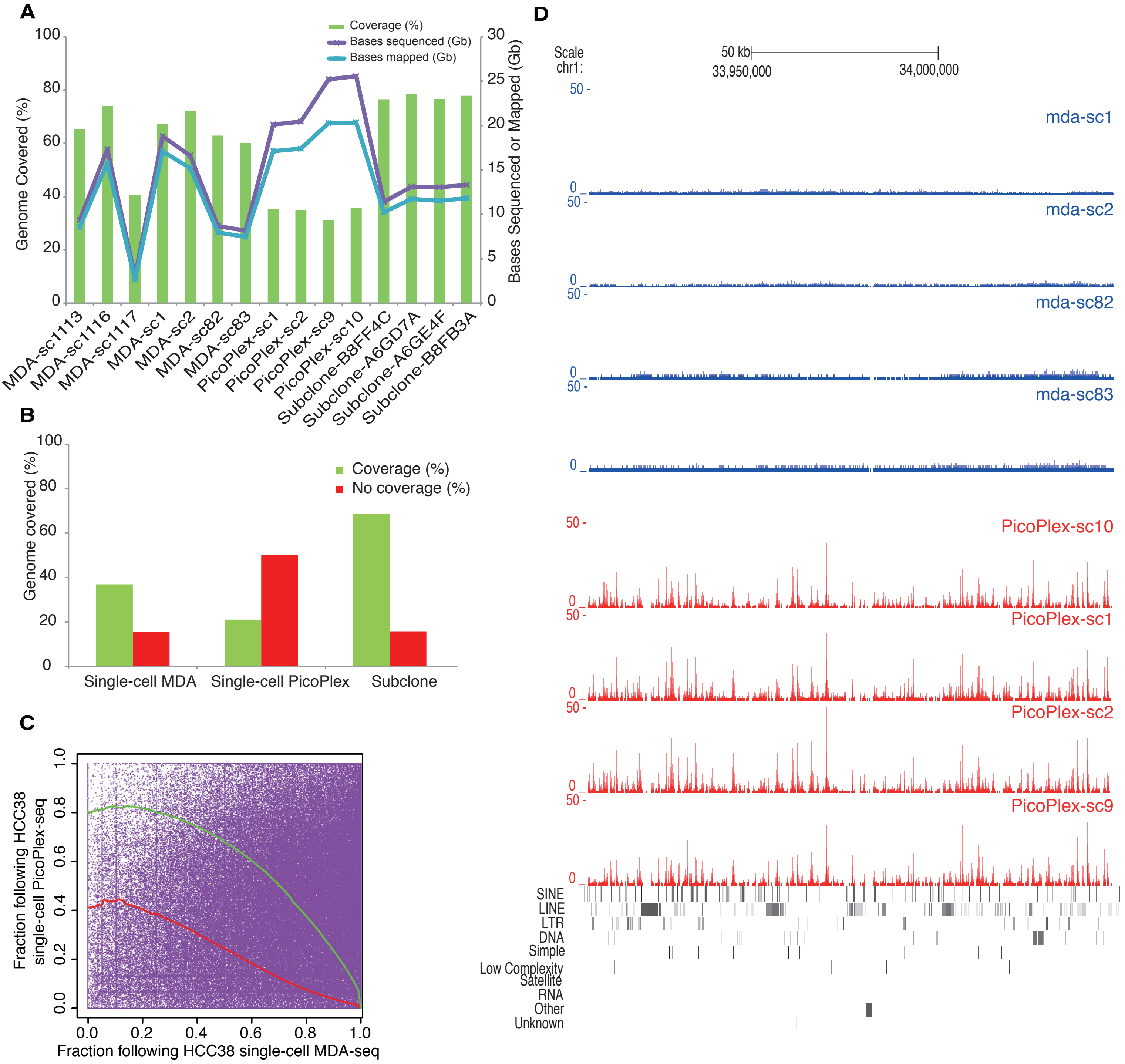

Supplementary Figure 1: Genome and exome coverage following single-cell WGA sequencing

(A) Fraction of the genome covered following single-cell WGA-sequencing. The X-axis lists the sequenced single-cell WGA-products and non-WGA subclone DNA-samples, the left Y-axis represents the fraction of the genome covered by reads with a minimum mapping quality of 30, the right Y-axis is the amount of Gb sequenced or mapped. Green color bars denote the percentage of the genome covered by at least 1 read. Although single-cell PicoPlex libraries were sequenced deeper (>20Gb) than single-cell MDA libraries (purple and blue lines), the obtained breadth of genomic coverage was poorer. (B) Fraction of the genome that is covered or missed recurrently following single-cell WGA-sequencing. The X-axis shows the single-cell WGA-sequencing methods in comparison with sequencing non-WGA DNA of the subclones. The Y-axis represents the fraction of the genome covered (green bars) or missed (red bars) recurrently across all WGAed single-cell genomes or three non-WGAed subclone genomes by reads with a minimum mapping quality of 30. Single-cell PicoPlex sequences recurrently miss more parts of the genome than single-cell MDA-sequences. (C) Fraction of the exome covered following single-cell WGA-sequencing. Each purple dot represents the fraction of an exome locus covered by either WGA-sequencing approach (X-axis: the average signal across 4 MDA-WGAed cells; Y-axis: the average signal across 4 PicoPlex-WGAed cells). The red line indicates the fraction of loci having a better coverage following single-cell PicoPlex-WGA sequencing. The green line indicates the fraction of loci having a better coverage following single-cell MDA-WGA sequence analysis. The exome loci coordinates are based on the Wellcome Trust Sanger Institute's exome pulldown design. (D) Visual representation of the sequence coverage (normalized to the amount of bases mapped) of a locus on chromosome 1 across 4 MDA-WGAed and 4 PicoPlex-WGAed single cells.

Supplementary Figure 2

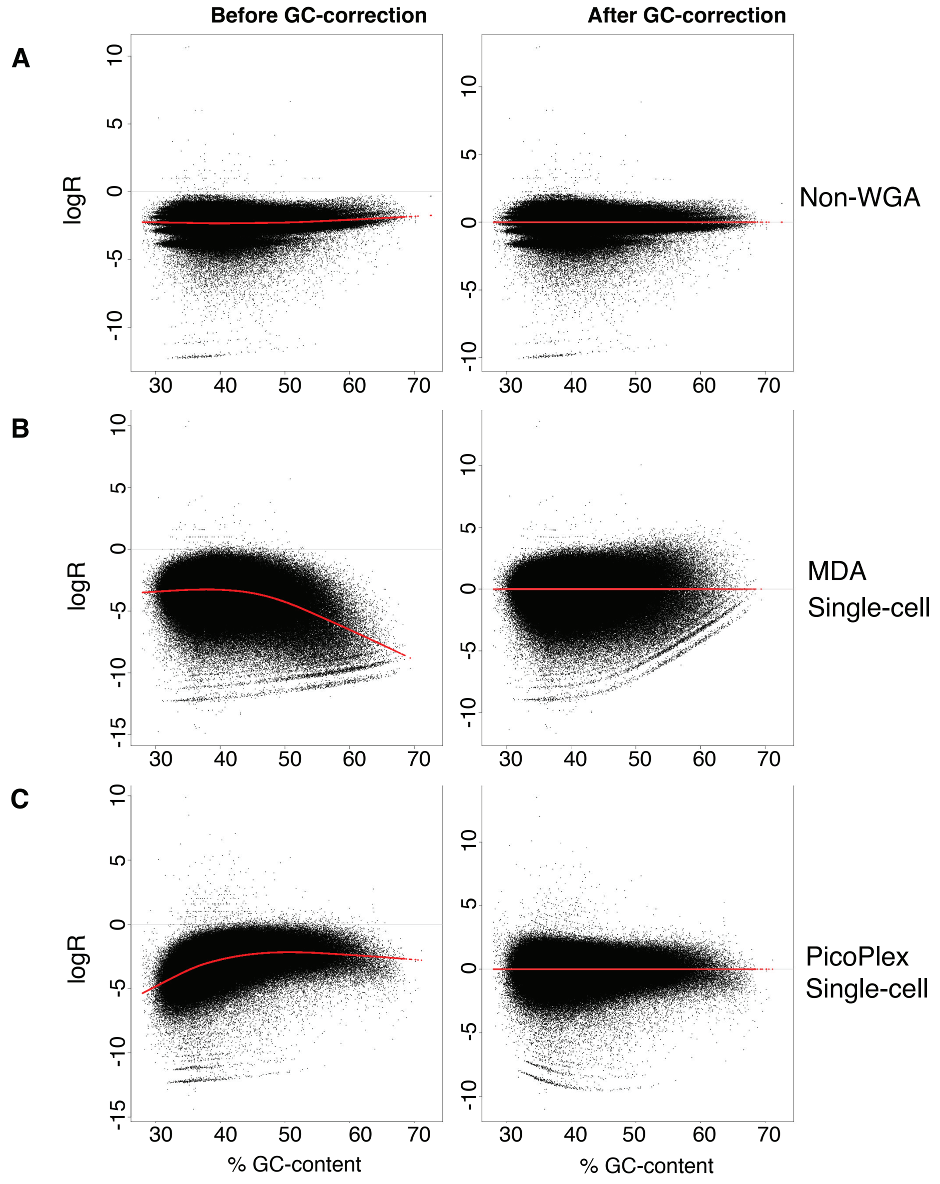

Supplementary Figure 2: Focal read-depth signals of WGAed single-cell genomes are affected by %GC-content of the locus
LogR ratios (Y-axis) of focal read-depth of the test sample versus the read-depth of a deep-sequenced normal blood non-WGA DNA-sample (Methods, 10kb genomic bins were applied for focal read-depth counting) depicted in the context of %GC-content (X-axis) of their respective genomic bin. The test sample is in (A) non-WGA DNA of the reference B8FF4C HCC38-subclone, in (B) an MDAed single-cell genome, in (C) a PicoPlex-amplified single-cell genome. LogR ratios before (left panels) and after (right panels) Loess fit normalization for %GC-content are shown.

Supplementary Figure 3

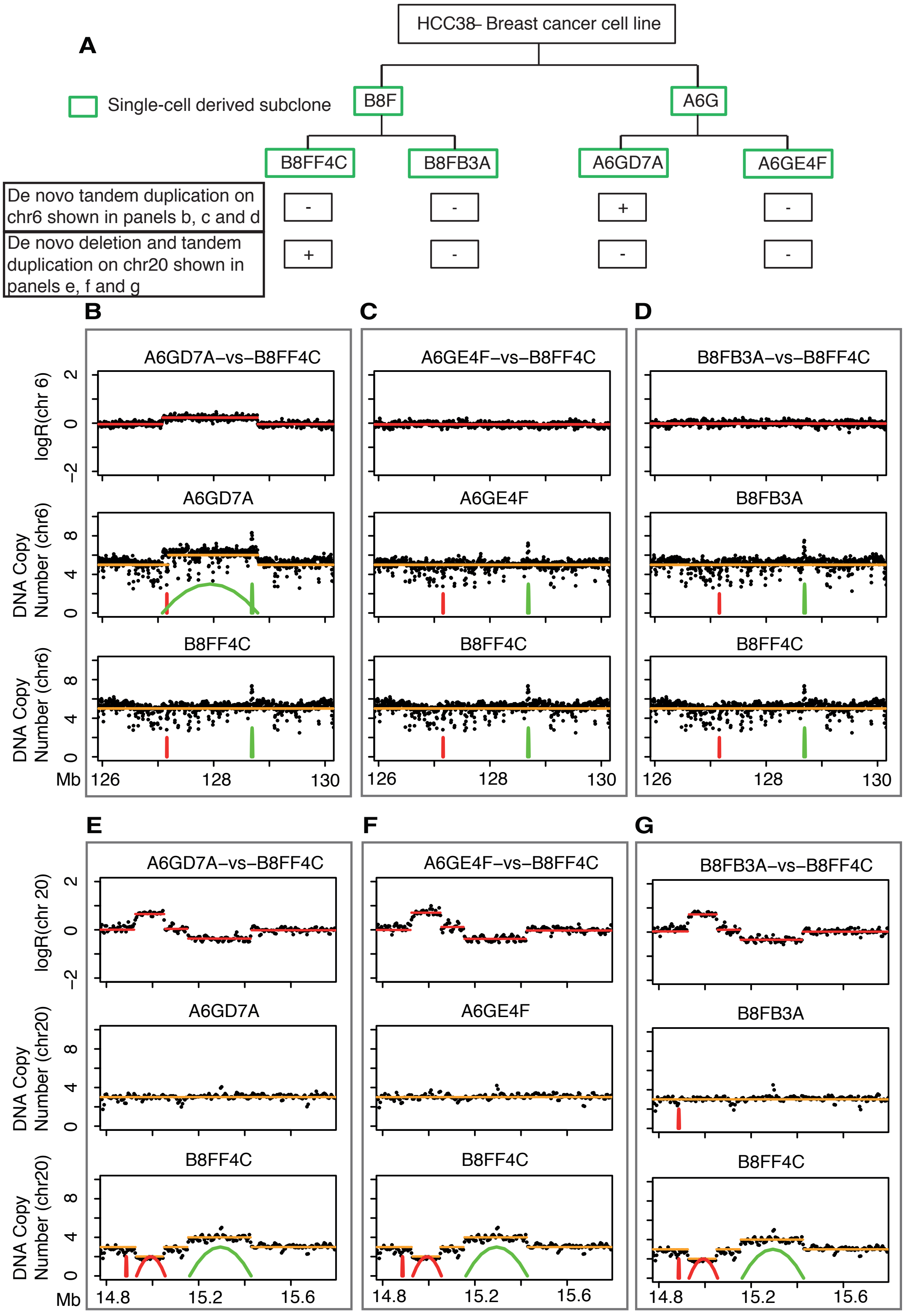

Supplementary Figure 3: The unstable genetic nature of HCC38 and evidence for ongoing tandem duplication events

(A) A tree of the single-cell derived subclones. A number of *de novo* aberrations that are unique amongst the subclones are indicated (+ for present, - for absent). (B-G) Evidence for the tandem duplication mutator phenotype in the breast cancer cell line HCC38. The top panel depicts the focal read-depth analysis of one subclone (A6GD7A, A6GE4F or B8FB3A) versus B8FF4C. The middle panel depicts the copy number profile of the first subclone (either A6GD7A, A6GE4F or B8FB3A). The lower panel shows the copy number landscape of the reference B8FF4C subclone. Groups of aberrantly mapping read-pairs for deletions (red arches) and tandem duplications (green arches) are shown in the middle and lower panel as well. (B-D) Subclone A6GD7A demonstrates a *de novo* tandem duplication not present in the other subclones (B8FF4C, B8FB3A, A6GE4F). (E-G) Subclone B8FF4C demonstrates a *de novo* tandem duplication and flanking deletion that are unique amongst the subclones.

Supplementary Figure 4

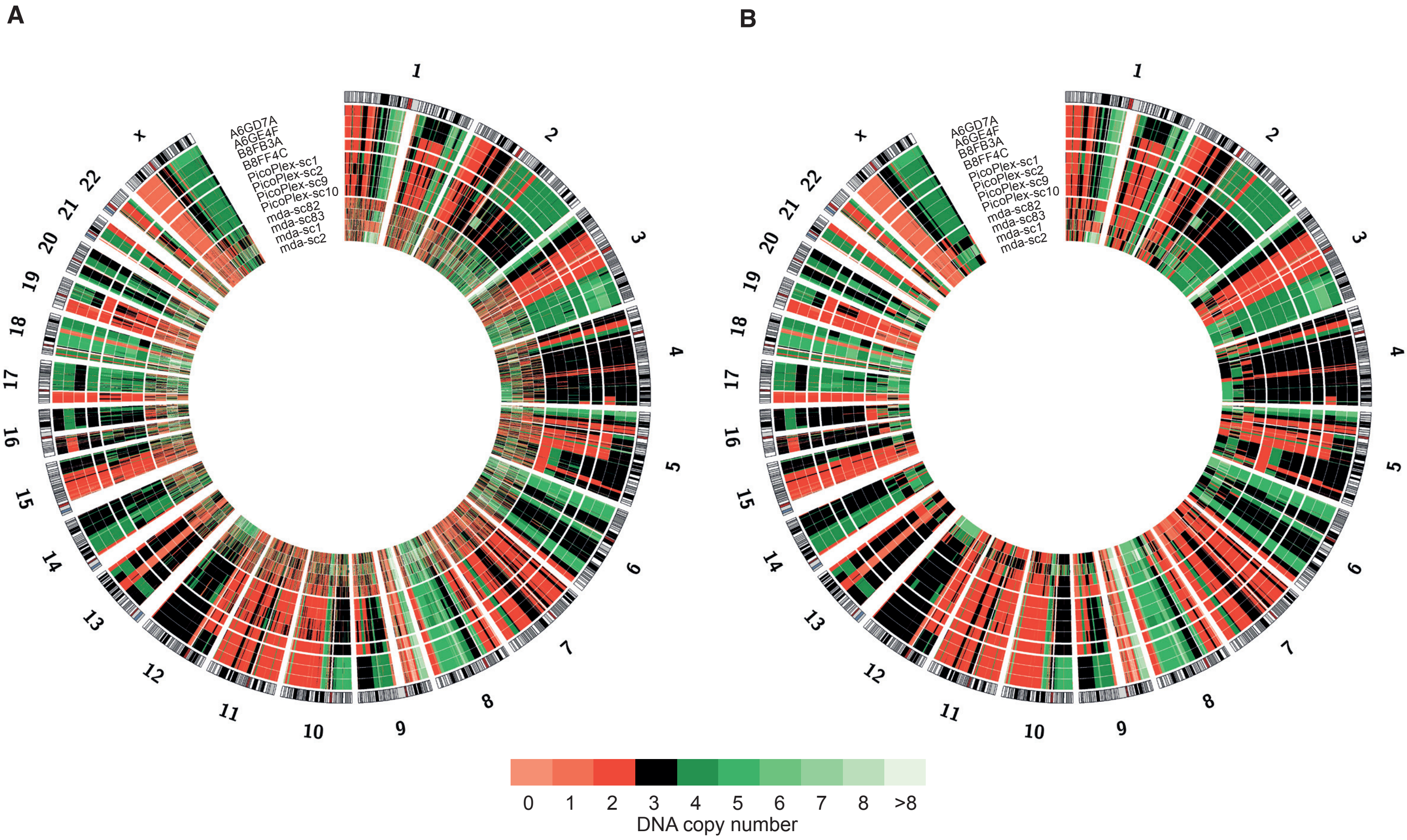

Supplementary Figure 4: Genome-wide integer DNA-copy number profiles for each multi-cell and single-cell tumor sample

By increasing the size of the bins for focal read-depth counting higher genome-wide copy number concordances with B8FF4C could be achieved. (A) Segments of integer DNA-copy number states following focal sequence-depth analyses using 10kb-bins and PCF segmentation (γ=25) across all autosomes and the X chromosome. (B) Segments of integer DNA-copy number states following focal sequence-depth analyses using 50kb-bins and PCF segmentation (γ=25) across all autosomes and the X chromosome. (A,B) The integer DNA-copy number is depicted as a heat map of which a color legend has been integrated in the figure. From the outside to the inside of the Circos-plot: the profiles of the non-WGA single-cell derived subclone samples (A6GD7A, A6GE4F, B8FB3A) and the reference B8FF4C sample are shown, followed by the 4 PicoPlex-amplified single cells (PicoPlex-sc1, PicoPlex-sc2, PicoPlex-sc9 and PicoPlex-sc10) and the 4 MDA-amplified single cells (mda-sc82, mda-sc83, mda-sc1, mda-sc2).

Supplementary Figure 5

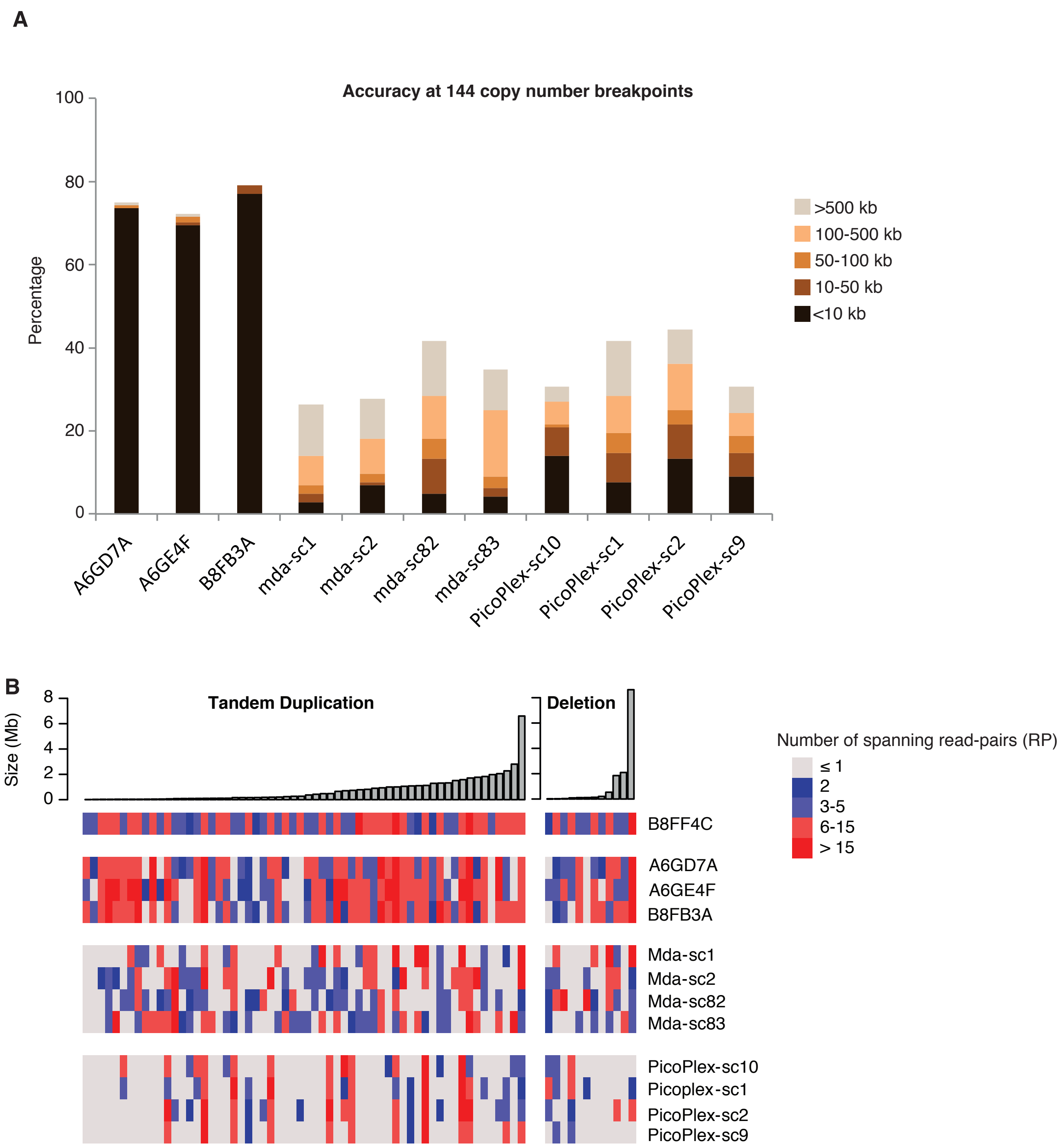

Supplementary Figure 5: Accuracy of copy number breakpoint detection

(A) Accuracy of copy number breakpoint detection following read-depth profiling. A set of 144 breakpoints of 72 DNA-copy number segments (for sizes of the segments see panel B), which were in addition corroborated by at least 2 discordantly mapping read-pairs, were selected from the focal sequence-depth analysis of the reference B8FF4C non-WGA DNA-sample. The breakpoints were aligned with the copy number profiles of all sequenced single cells and three non-WGAed HCC38 subclones (A6GD7A, A6GE4F or B8FB3A). Only data of those single-cell or subclone DNA-segments that had a copy-number match with the selected B8FF4C segments are depicted. It is clear that single-cell PicoPlex sequencing delivers more accurate breakpoints than single-cell MDA sequencing based on focal read-depth analysis. (B) Groups of discordantly mapping read pairs that support the 72 B8FF4C copy number segments. The top panel indicates the size distribution of the selected 72 DNA copy number segments of B8FF4C. The color code indicates the amount of discordantly mapping read-pairs supporting the reference DNA-copy number segment in the single-cell WGA or subclone non-WGA sequence.

Supplementary Figure 6

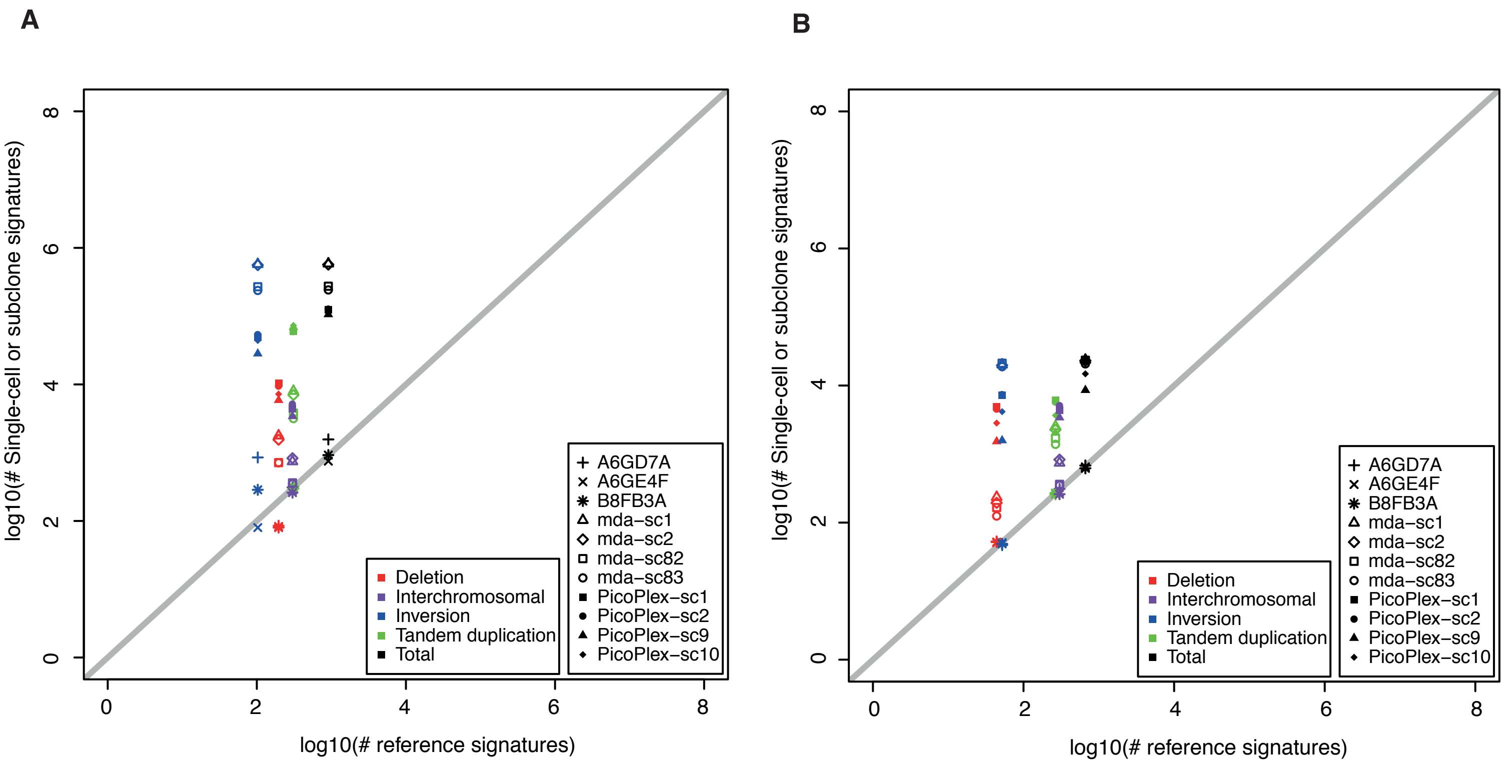

Supplementary Figure 6: Amount of rearrangement signatures in the paired-end map

(A) The amount of signatures spanned by 2 or more discordantly mapping read-pairs in the reference B8FF4C sequence (X-axis) as well as in the single-cell WGA or non-WGA subclone DNA-sequences (Y-axis) are indicated. Signatures for deletions, tandem duplications, inversions and interchromosomal events are shown in red, green, blue and purple, respectively. For each sample there is a unique symbol. The total amount of signatures is depicted in black. (B) Identical to (A) but the discordantly mapping read-pairs in addition had to encompass a region of at least 5 kilobases (except for signatures typical of interchromosomal rearrangements).

Supplementary Figure 7

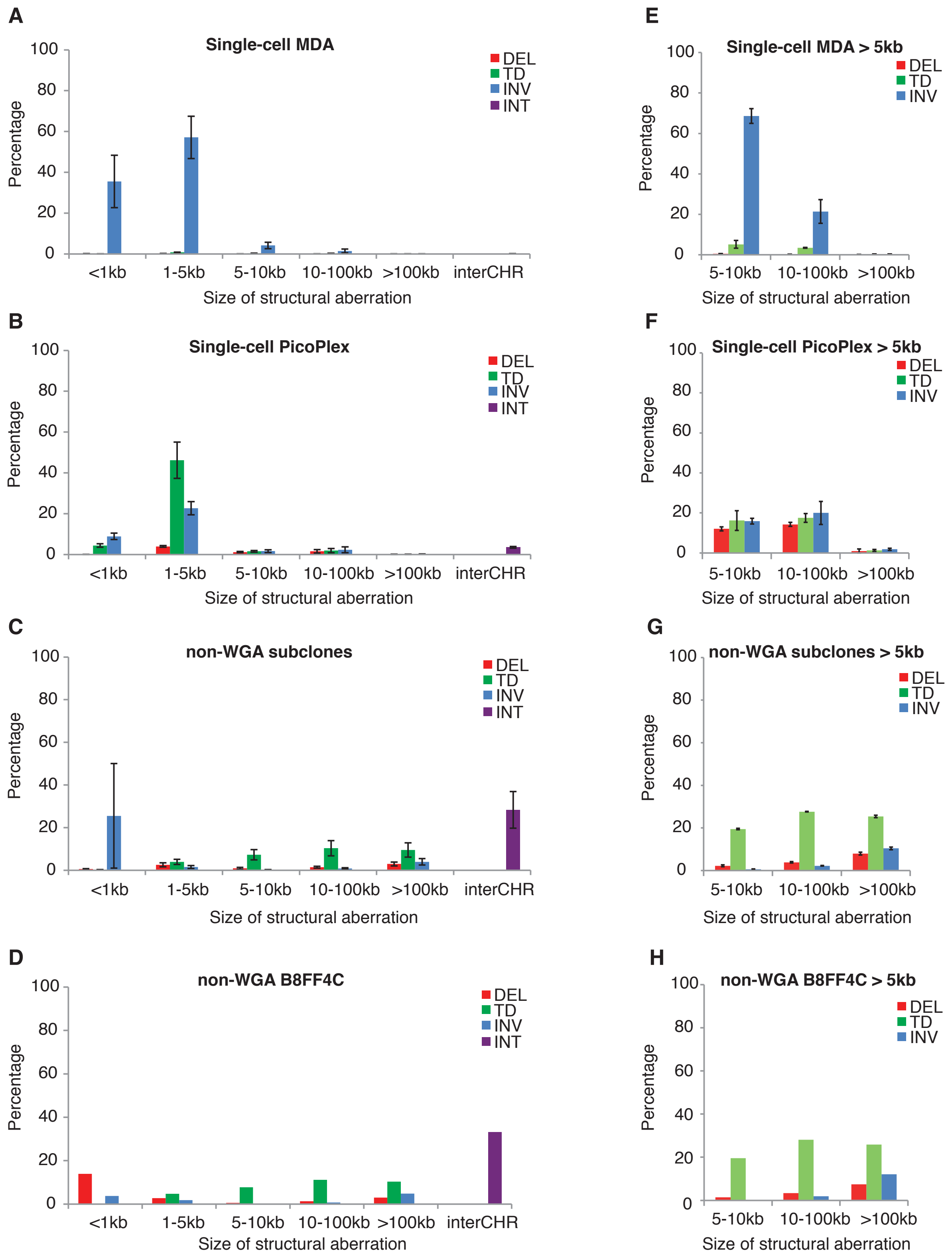

Supplementary Figure 7: Single-cell WGA contaminates maps of genomic structure with read-pair WGA-artefacts
(A-D) The fractions of the total amount of rearrangement signatures (%), which are supported by two or more discordantly mapping read-pairs in the respective paired-end map, are shown subdivided according to rearrangement type and size. Data for deletions, tandem duplications, inversions and interchromosomal rearrangements is depicted in red, green, blue, and purple respectively. The panels indicate the distribution of these fractions in the paired-end maps of (A) MDA-WGAed single cells, (B) PicoPlex-WGAed single cells, (C) non-WGAed subclones and (D) the non-WGAed B8FF4C reference. The amount of signatures supported by two or more aberrantly mapping read-pairs is shown in Supplementary Figure 6A.(E-H) Fractions of the rearrangement signatures (%) supported by 2 or more aberrantly mapping read-pairs and encompassing more than 5 kb in the respective paired-end maps are shown. Data for deletions is depicted in red, tandem duplications in green, inversions in blue. The panels indicate the distribution of these fractions in the paired-end maps of (E) MDA-WGAed single cells, (F) PicoPlex-WGAed single cells, (G) non-WGAed subclones and (H) the non-WGAed B8FF4C reference. The amount of signatures corroborated by two or more aberrantly mapping read-pairs and encompassing more than 5 kb is shown in Supplementary Figure 6B.

Supplementary Figure 8

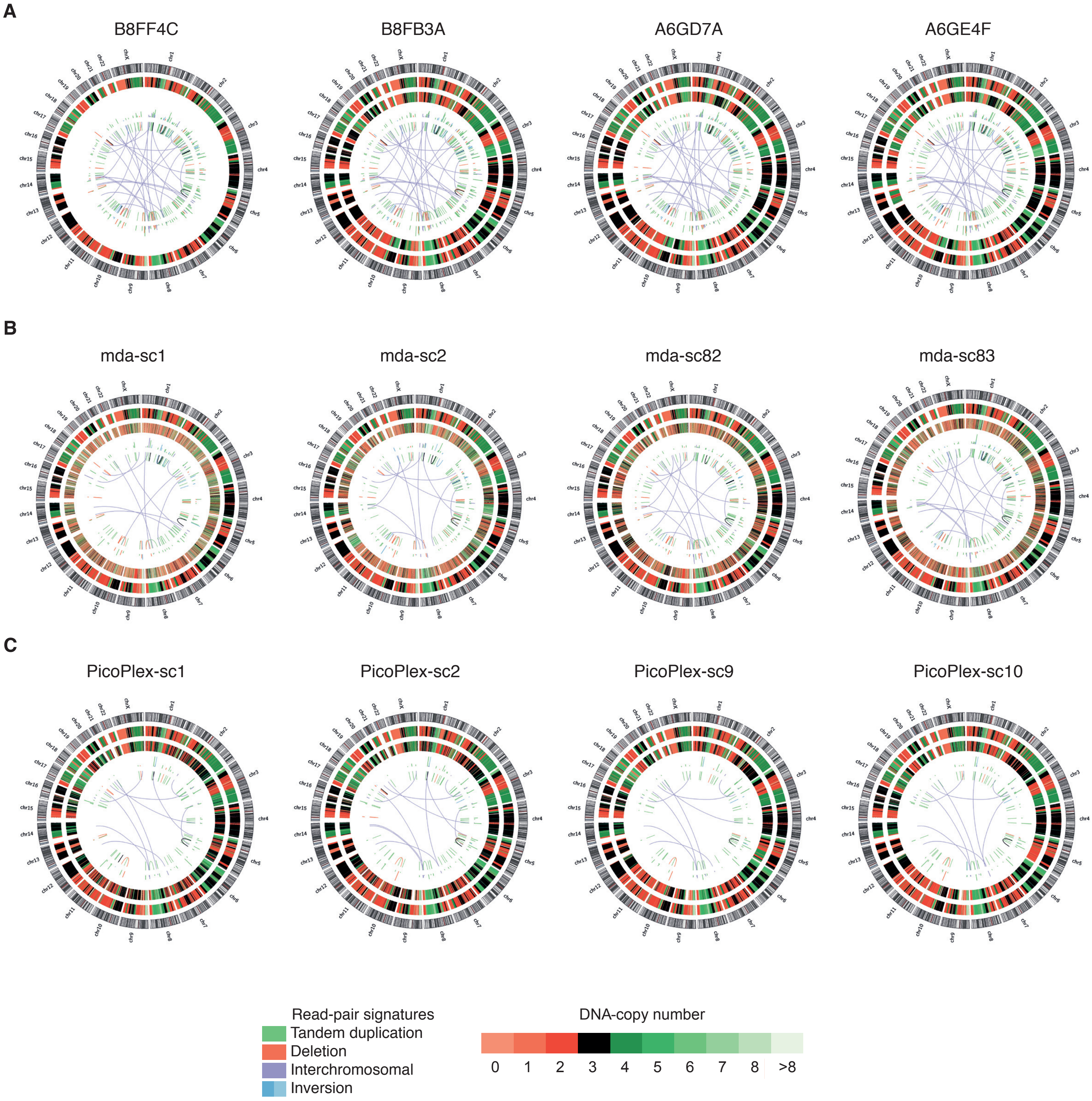

Supplementary Figure 8: Sensitivity of single-cell paired-end maps

(A-C) Circos-plots depict confirmed HCC38-rearrangements (centre of the plot) that are identified in the non-WGA subclone and the single-cell paired-end maps. Each Circos-plot also depicts the integer DNA-copy number heat map (using 10 kb-bins and γ=25) of the non-WGA B8FF4C (heat map on the outer circle) and of the specific subclone or single-cell sample (heat map on the inner circle). The plot for the B8FF4C reference subclone shows only its own copy number heat map. Color legends for the rearrangements and the copy number heat maps are indicated. (A) Circos-plots for the reference sample and the non-WGA subclones from left to right: B8FF4C (reference), B8FB3A, A6GD7A and A6GE4F. (B) Circos-plots for MDA-amplified single cells from left to right: mda-sc1, mda-sc2, mda-sc82 and mda-sc83. (C) Circos-plots for PicoPlex-amplified single cells from left to right: PicoPlex-sc1, PicoPlex-sc2, PicoPlex-sc9 and PicoPlex-sc10.

Supplementary Figure 9

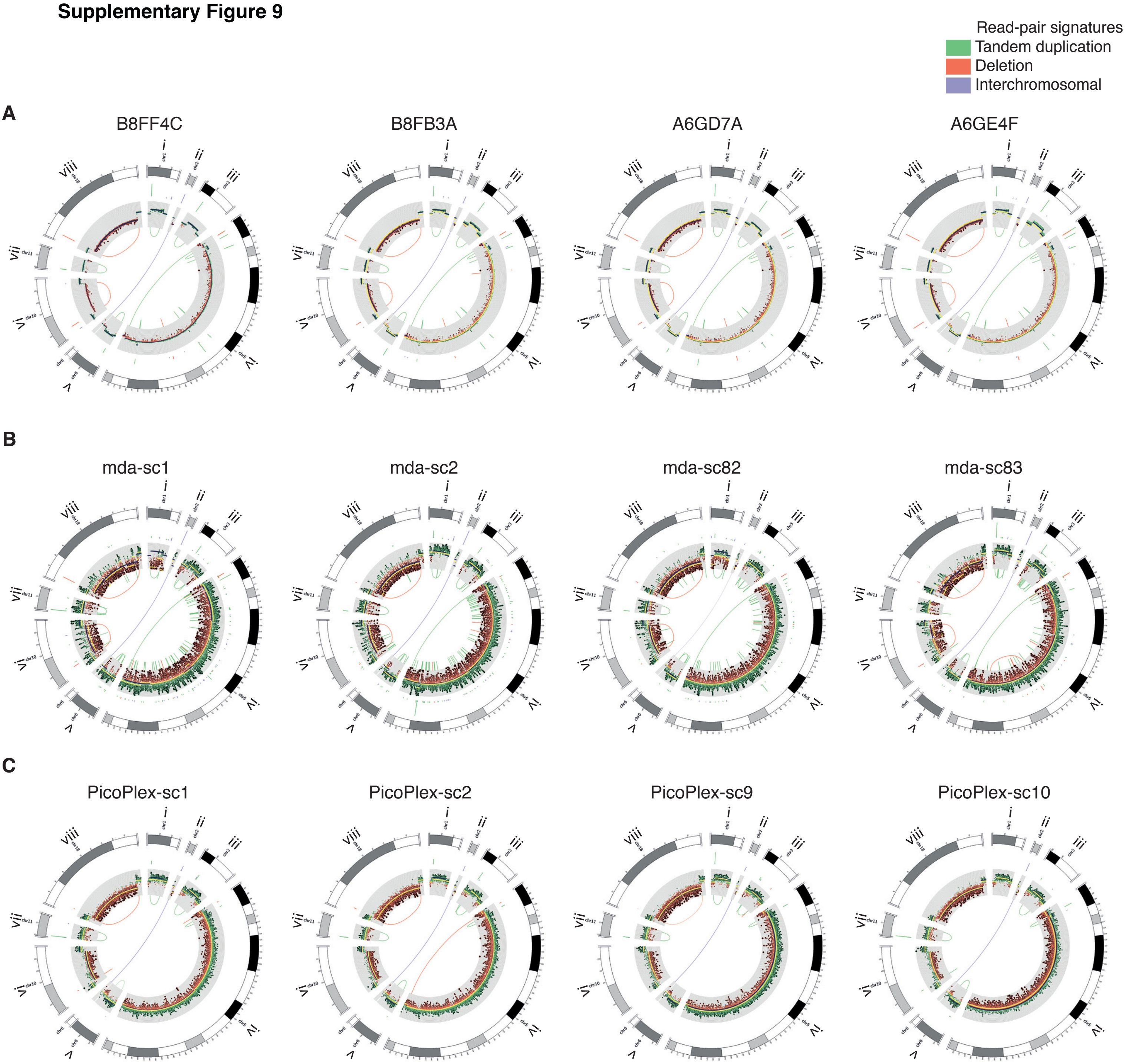

Supplementary Figure 9: Detection of imbalanced structural variants by (single-cell) paired-end mapping: all non-WGA and single-cell samples are depicted for the selected rearrangements shown in Figure 3

Integration of focal read-depth anomalies with aberrantly mapping read-pairs allows accurate copy number variant detection in single cells and discloses the structure of the DNA-imbalance. Read-pair signatures typical for tandem-duplications, deletions or interchromosomal lesions are depicted in the centre of the Circos-plot in green, red and purple respectively. The amount of read-pairs supporting each rearrangement is depicted by a bar (scale 2 to 30) at the start of each rearrangement signature in the outer circle of the Circos-plot. Subsequently, the logR-values are shown on a grid (logR-values above zero are depicted in green, below zero in red). Dark blue lines depict the B8FF4C reference logR-segments determined from sequences of a non-WGA DNA-sample, yellow lines indicate the sample's logR-segments (segmentation penalty γ=150). The Circos-plots show for all non-WGA subclone-samples (A), all single-cell MDA-samples (B) and all single-cell PicoPlex-samples (C) the selected rearrangements of main Figure 3. The depicted rearrangements (i-viii) are described in more depth in the legend of Figure 3. For the single-cell PicoPlex samples, only aberrantly mapping read-pairs typical for deletions, tandem duplications and interchromosomal rearrangement are shown which are captured from the refined pool of aberrantly mapping read-pairs using a ~50 kb radius around the single-cell PicoPlex logR breakpoints. Faded rearrangements (PicoPlex-sc1 rearrangement 'v'; PicoPlex-sc9 rearrangement 'viii') have a read-pair count of 1. For the interchromosomal rearrangement between chromosomes 2 and 6 (rearrangement 'ii') a minimum read-pair count of 9 was applied, if this threshold was not reached a faded purple line represents the rearrangement.

Supplementary Figure 10

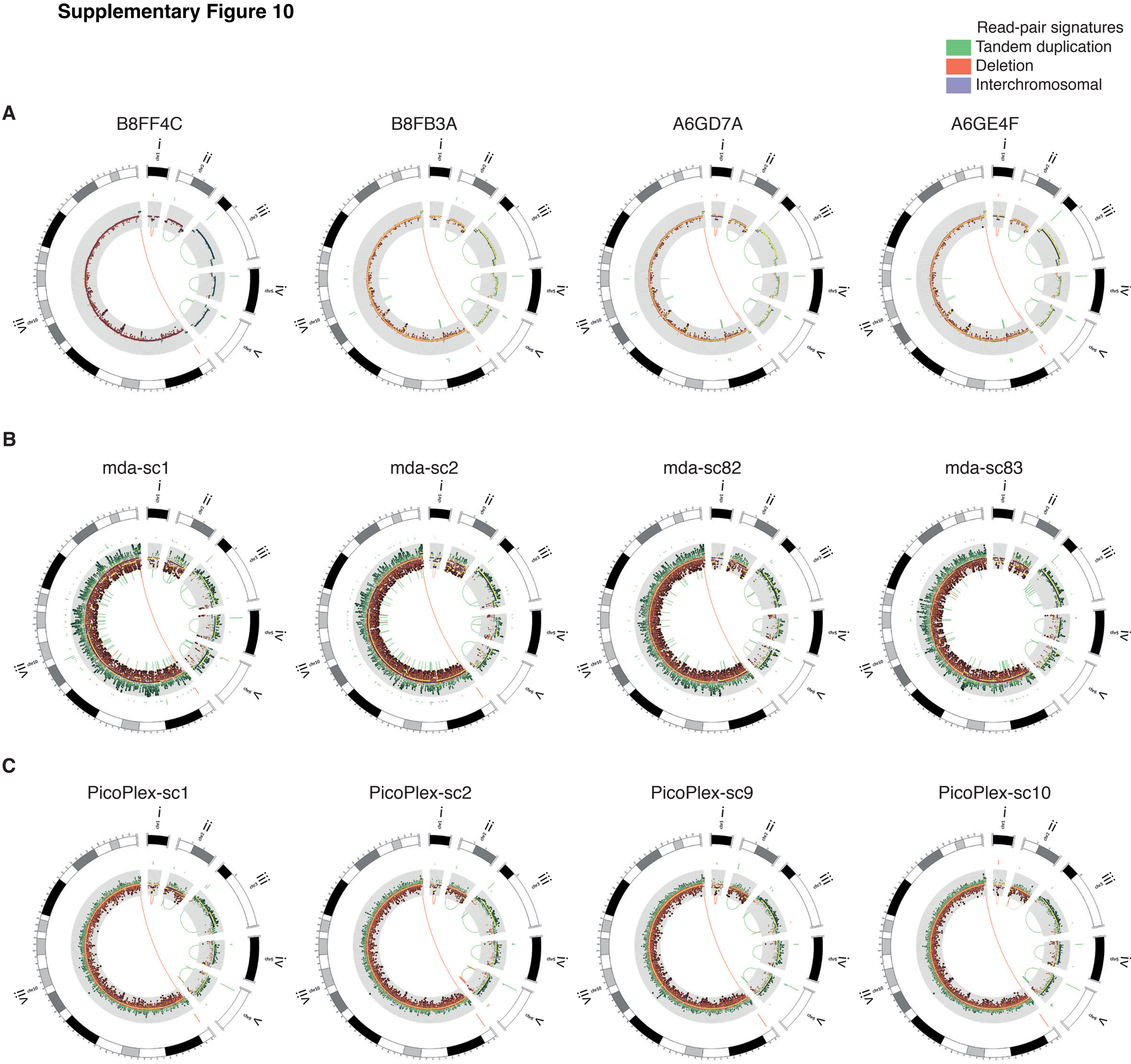

Supplementary Figure 10: Detection of imbalanced structural variants by (single-cell) paired-end mapping: all non-WGA and single-cell samples are depicted for the selected rearrangements shown in Figure 4

For the rearrangements shown in main figure 4, the Circos-plots for all non-WGA subclone-, single-cell MDA- and PicoPlex-samples are shown in panels (A), (B) and (C) respectively. For the single-cell PicoPlex samples, aberrantly mapping read-pairs typical for tandem-duplication (green), deletion (red), and interchromosomal rearrangement (purple) signatures were captured from the refined pool of aberrantly mapping read-pairs using a ~50 kb radius around single-cell PicoPlex logR breakpoints. Furthermore, for intra-chromosomal rearrangements, only those encompassing more than 5 kb are depicted. In PicoPlex-sc10 the discordant read-pair signature was present for rearrangement ‘i’, yet not captured by baiting as the logR-segmentation missed the deletion in this cell (shown by a faded red line). The amount of read-pairs supporting each rearrangement is depicted by a bar (scale 2 to 30) at the start of each rearrangement signature in the outer circle of the Circos-plot. Subsequently, the logR-values are shown on a grid (logR-values above zero are depicted in green, below zero in red). Dark blue lines depict the B8FF4C reference logR-segments determined from sequences of a non-WGA DNA-sample, yellow lines the sample’s logR-segments. Faded rearrangements (mda-sc2 rearrangement ‘i’; mda-sc82 rearrangement ‘v’; PicoPlex-sc10 rearrangement ‘ii’) have a read-pair count of 1.

Supplementary Figure 11

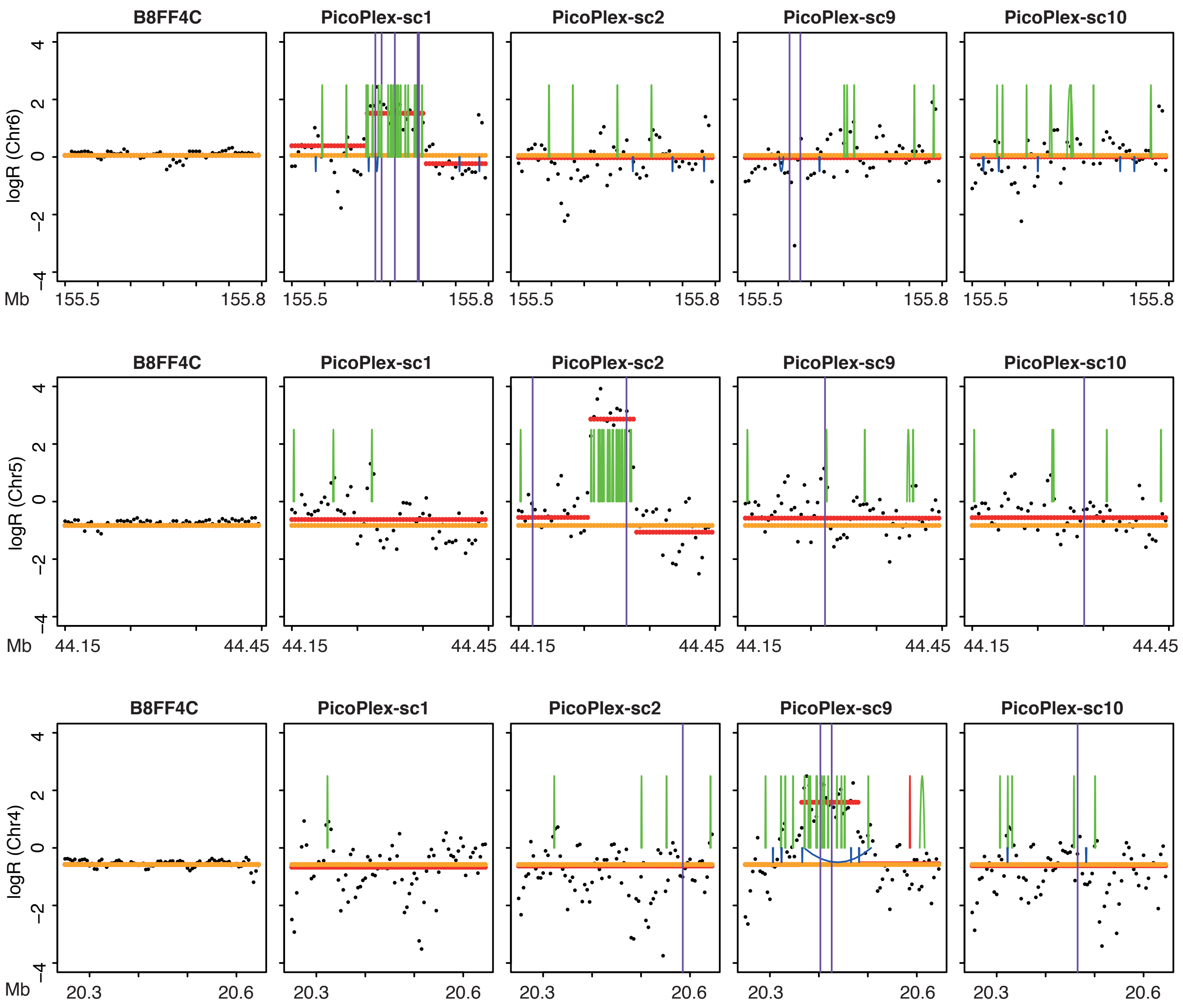

Supplementary Figure 11: Paired-end mapping identifies PicoPlex WGA-pileup artefacts

Focal read-depth analysis of single-cell PicoPlex-amplified genomes (red segmentation) disclosed putative DNA-gains (see cell 'PicoPlex-sc1' in the top panels; cell 'PicoPlex-sc2' in the middle panels; cell 'PicoPlex-sc9' in the bottom panels) that were not recurrent in the other cells nor in the reference profile (orange segmentation). Integration with data of aberrantly mapping read-pairs revealed that these loci were densely coated with multiple small tandem duplications (green arches) instead of a single tandem duplication signature that connects both breakpoints of the putative DNA-gain. Hence, we interpreted such *de novo* copy number changes to be WGA-artefacts. Signatures for inversion and interchromosomal rearrangement events are depicted in blue and purple respectively. Each left panel shows the data of the non-WGA reference subclone B8FF4C. The following four panels show single-cell PicoPlex sequence results.

Supplementary Figure 12

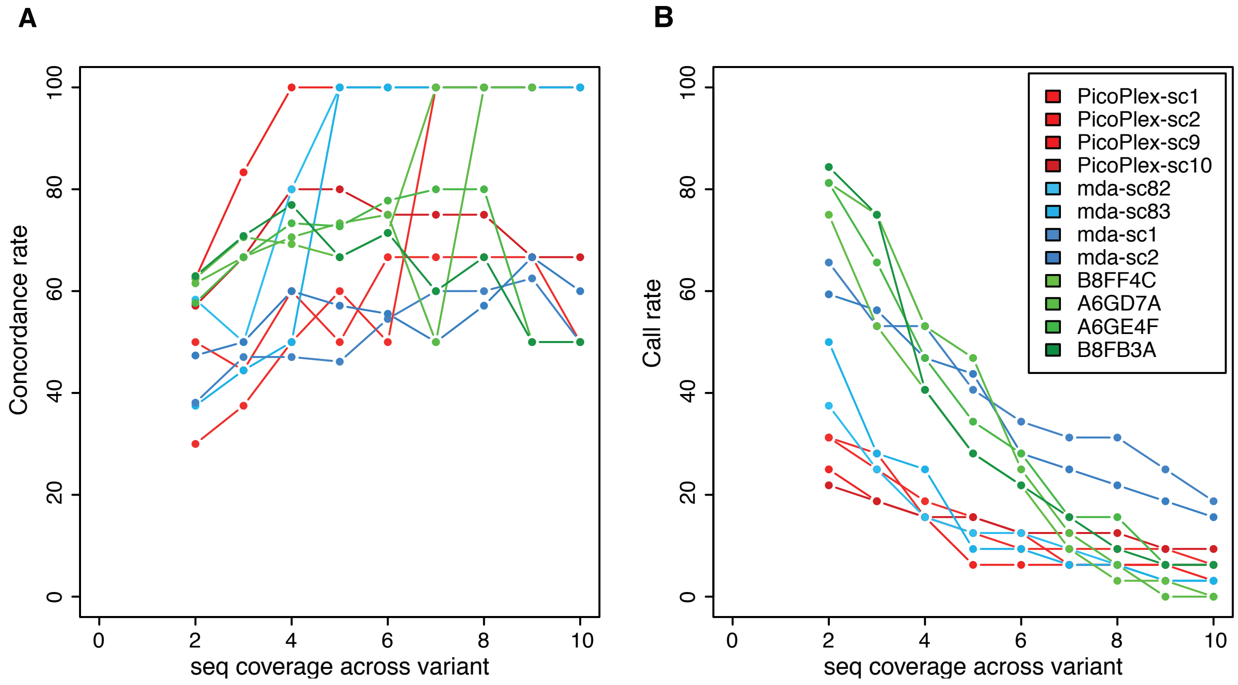

Supplementary Figure 12: Detection of base mutations in single-cell sequences

(A,B) Genotyping of confirmed heterozygous HCC38 somatic mutations in the single-cell or subclone sequences. (A) Concordance of the mutation's zygosity with the HCC38 profile (Y-axis) in function of the read coverage across the mutation (X-axis) in the single-cell or subclone sequences. (B) Call rate of the somatic mutations (Y-axis) in function of the read-depth across the mutations (X-axis) in the single-cell or subclone sequences. Results for single-cell PicoPlex sequences, single-cell MDA sequences and non-WGA subclone sequences are indicated in red, blue and green colors respectively.

Supplementary Figure 13

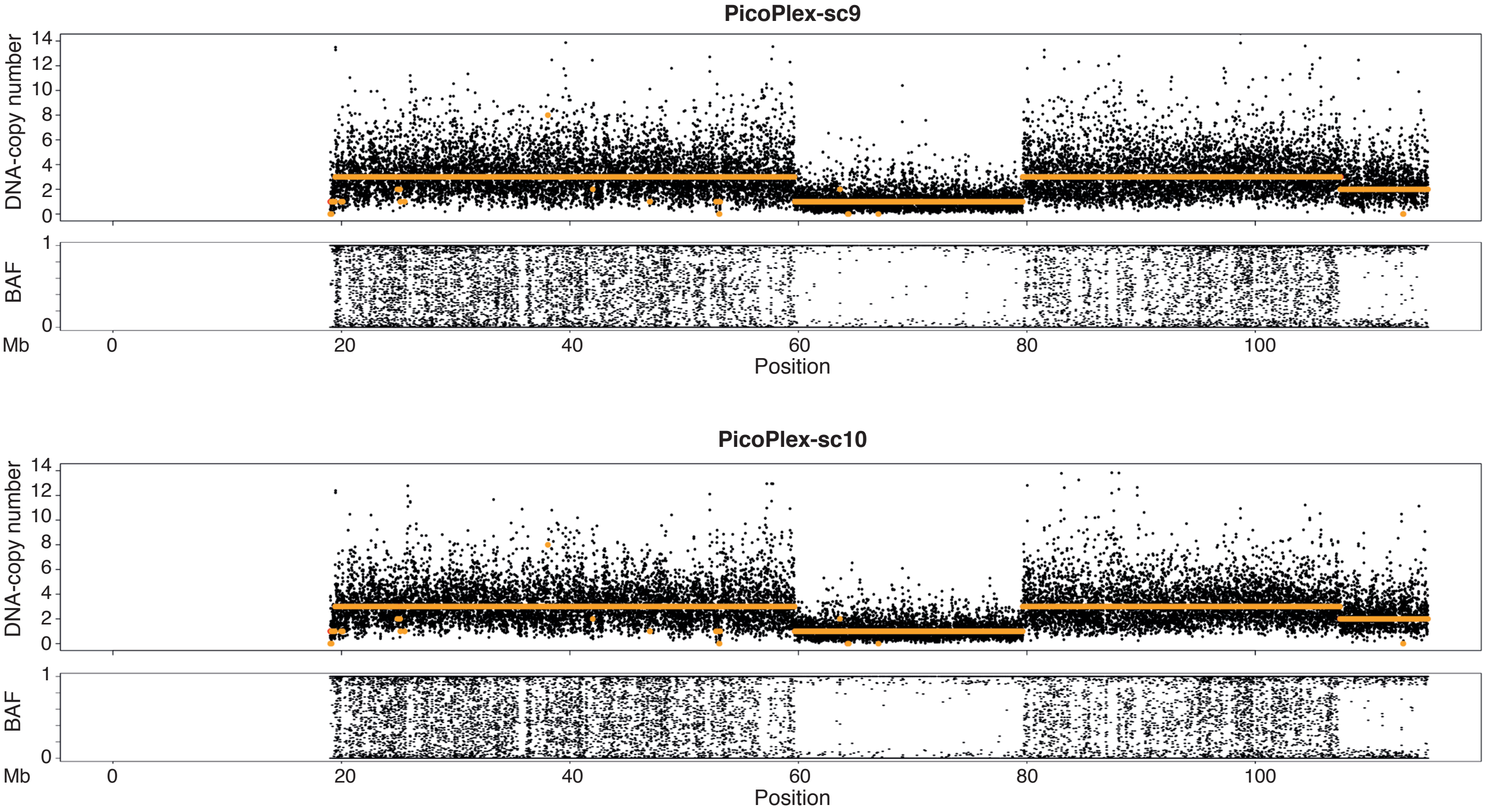

Supplementary Figure 13: SNP B-allele fractions computed from single -cell PicoPlex sequences corroborated DNA - imbalances

The single-cell DNA-copy number and SNP B-allele fraction (BAF) landscape of chromosome 13 in sister cells 'PicoPlex-sc9' and 'PicoPlex-sc10'. Orange lines representing the B8FF4C copy number segments (10kb-bin and γ=25) are overlaid on top of the red lines which represent the single-cell DNA copy number segments (10kb-bin and γ=50). It is clear that for this chromosome the DNA-losses are corroborated by loss-of-heterozygosity detection in the BAF. Cell 'PicoPlex-sc9' is shown in the top panel, cell 'PicoPlex-sc10' in the bottom panel.
